# Endohedral [Au@In_10_]^9–^ Cluster: Synthesis and Characterization of Na_3+*x*_ *A*_6–*x*_In_10_Au (*x* = 0, 0.25; *A* = Rb, Cs)

**DOI:** 10.1021/acs.inorgchem.5c04619

**Published:** 2026-01-30

**Authors:** Melissa Janesch, Florian Pielnhofer, Michal Dušek, Ilya G. Shenderovich, Stefanie Gärtner

**Affiliations:** † Institute of Inorganic Chemistry, 9147 University of Regensburg, Universitätsstr. 31, 93053 Regensburg, Germany; ‡ Institute of Physics of the Czech Academy of Sciences, Na Slovance 2, 182 21 Prague 8, Czech Republic; § Central Analytics, 9147 University of Regensburg, Universitätsstr. 31, 93053 Regensburg, Germany

## Abstract

The synthesis and characterization of two new compounds Na_3_Rb_6_In_10_Au and Na_3.25_Cs_5.75_In_10_Au are reported, which contain [Au@In_10_]^9–^ clusters as anionic entities. Single-crystal X-ray structure analysis shows that the alkali metal composition is the key factor for structure formation, while the anionic entity remains unchanged. The chemical composition was confirmed by SEM/EDS measurements, and the given compositions of both compounds are fixed according to the line compounds. Quantum chemical calculations for the compound Na_3_Rb_6_In_10_Au were performed and show a band gap at the Fermi level, classifying the materials as salt-like, including endohedral [Au@In_10_]^9–^ *Zintl-*type clusters. Dissolution experiments in liquid ammonia were carried out, revealing In_2_Au as the reaction product.

## Graphical abstract

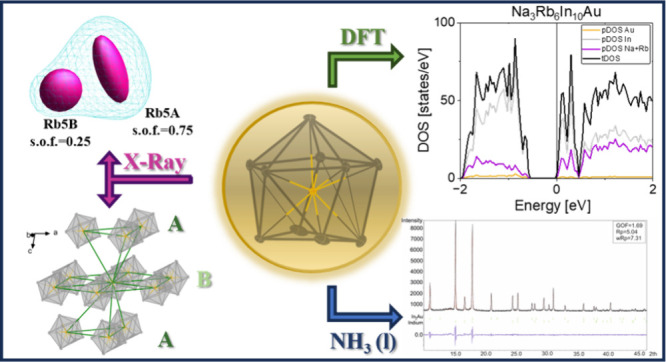

## 1 Introduction

Endohedral clusters have attracted considerable theoretical and experimental attention in inorganic chemistry due to their remarkable electronic flexibility.^1,2^ Numerous examples of such cluster phases arise from combinations of electropositive metals with indium or thallium. In related ternary phases with a low gold content, the underlying cluster topology is generally retained.^3^ Larger quantities have been extensively studied and show a broad structural variety, which reaches from isolated clusters^4–8^ over two-dimensional layers^9–17^ to three-dimensional networks.^18–25^ Here, intercluster bonding reduces the charge of the anionic entities and stabilizes these structural features. In general, the alkali metal indium system^26–43^ bridges the structural properties of both the lighter alkali metal gallides with the two- or three-dimensional networks^44–54^ and the alkali metals thallides, forming preferably isolated clusters.^55–60^

To enhance the stability of the anionic cluster entities and lower the high charges of isolated, empty clusters, the incorporation of interstitial atoms is still proven to be a powerful tool, too.^61^ These endohedral clusters are known in the solid state but also can be crystallized from solution and are stabilized by bulky metal organic ligands.^1,62–65^ This is displayed in many ternary alkali metal trielide systems, for example, in endohedral [*M*@*Tr*_10_]^*x*−^ (*Tr*= Ga–Tl; *x* = 8, 10) clusters, which are observed in K_8_[Zn@*Tr*_10_] with (*Tr* = Ga, In, Tl),^66,67^ K_10_[*M*@In_10_] with *M* = Ni, Pd, Pt^68^ as well as in Na_10_[Ni@Ga_10_].^69^ The total valence electron count of all *A*_*x*_[*M*@*Tr*_10_] (*A* = Na–Cs, *M* = Ni, Pd, Pt, Zn, *Tr* = Ga–Tl; *x* = 8, 10) sums up to 50 electrons, and a pseudo band gap reflects their salt-like character. The replacement of the transition metal by gallium results in metallic *A*_8_[Ga@Tl_10_] (*A* = K–Cs).^70^ In contrast, the substitution of one indium by one mercury position in K_8_In_10_Hg does not yield an endohedral cluster, but results in a closed shell [In_10_Hg]^8–^ cluster,^71^ which is isoelectronic to [In_11_]^7–^.^41^

The ten-atomic clusters referred to as centaur polyhedra are well-known structural features in intermetallic compounds and can be described as a fusion of a cube and an icosahedron or a distorted 4-fold capped trigonal prism.^72–74^ In intermetallic compounds, the centaur polyhedron is filled by an endohedral atom.^75^ Recently, this type of cluster was also observed for group 14 elements.^76–78^ Here, the centaur polyhedra [*Tt*_10_] [*Tt* = Ge, Sn] are empty but stabilized by bulky ligands.

In this context, we investigated the incorporation of coinage metals in indium clusters, for which no such compounds have been reported yet. In the literature, the only evidence within this class of materials was reported in the case of copper, but no structure was determined.^68^ Further, calculations predict the existence of ternary alkali metal–indium phases with an interstitial coinage metal, but this has not yet been proven experimentally.^79^

Here, we report on the preparation and characterization by single-crystal and powder X-ray diffraction and SEM-EDS measurements of the two compounds Na_3_Rb_6_In_10_Au (**1**) and Na_3.25_Cs_5.75_In_10_Au (**2**), which both include molecular, endohedral [Au@In_10_]^9–^ indium clusters. Besides dissolution experiments in liquid ammonia, density functional theory (DFT) calculations for (**1**) and ^23^Na solid-state NMR spectroscopy were also carried out.

## 2 Experimental Section

### 2.1 Materials

Sodium (purity 99%, under mineral oil, Merck/Sigma-Aldrich, Darmstadt) was segregated for purification. Rubidium and cesium were obtained by reduction of RbCl or CsCl, respectively, with calcium and afterward purified by two times distillation.^80^ Indium drops (purity 99.99%, ABCR) and gold wire (purity 99.997%, Merck) were used without further purification and were stored under an inert gas atmosphere. Appropriate safety clothing, such as a lab coat, safety glasses, visors, and leather gloves, was worn during work in the laboratory. As a precaution, sand and appropriate fire extinguishers were placed near the distillation apparatus in case the apparatus should crack. After segregation or distillation, respectively, the sealed alkali metal ampules were stored in a sand bath in a fire-proof drawer and then, on demand, transferred into the glovebox, where they are opened.

### 2.2 Preparation

Due to the fact that the alkali metal indides are very sensitive toward air and moisture, all operations are performed under an inert gas atmosphere in a glovebox (Labmaster 130 G, Fa. M. Braun, Garching, Germany). For the synthesis of Na_3+*x*_ *A*_6–*x*_In_10_Au (*x* = 0, 0.25; *A* = Rb, Cs), the elements in their solid form (cesium was cooled down to obtain the solid form) were placed in a tantalum ampule (length: 3 cm, diameter: 1 cm), which was sealed under an argon atmosphere. The sealed ampules were placed in quartz glass tubes (QSIL GmbH, Ilmenau, Germany) and sealed again under an argon atmosphere. These sealed ampules were then placed in a tube furnace using the following temperature program: heating from room temperature to 973.15 K with a heating rate of 100 K/h, holding for 48 h, then cooled with a cooling rate of 3 K/h to room temperature. The role of the temperature program was also explored for both compounds: Three different cooling rates were tested out: (1) slow cooling as described above, (2) the sample was quenched in water, or (3) the sample was taken out of the furnace at 973.15 K, cooled to room temperature, and subsequently annealed for 5 days at 523.15 K and cooled to room temperature with 3 K/h. All three temperature programs yielded the compounds with the chemical composition Na_3_Rb_6_In_10_Au or Na_3.25_Cs_5.75_In_10_Au (see SI Chapter 5); only the crystal quality suffered after quenching compared to slow cooling.

### 2.3 X-ray Single-Crystal Analysis

A small number of crystals were transferred into vacuum-dried mineral oil. A suitable crystal was selected and mounted on a Rigaku XtraLAB Synergy R, DW diffractometer (Rigaku Polska sp. Z. o. o. UI, Wroclaw, Poland) (rotating anode X-ray tube, MoKα radiation, λ = 0.71073 Å; HyPix-Arc 150 detector) using MiTeGen loops for compound (**1**). The data were collected at 123 K and at 100 K as well. The data collection for compound (**2**) was carried out at 100 K on a Rigaku SuperNova diffractometer (X-ray: Mo/Ag microfocus, Atlas S2 detector). Crystallographic details for both compounds can be found in the SI Chapters 1–4.

For data collection and data reduction, CrysAlisPro (Version 171.44_32.117a) was used.^81^ The structure solution was carried out with ShelXT,^82^ and for the subsequent data refinement, ShelXL^83^ was applied. For visualization purposes, Olex2 was used, and the software Diamond4 ^84^ was chosen for the representation of the crystal structure. All atoms are depicted as ellipsoids with a 50% probability level.

For Na_3_Rb_6_In_10_Au, residual electron density was present around the [Au@In_10_]^9–^ clusters, which was not affected by the lower temperature measurement at 100 K. The same was observed for the heavier compound Na_3.25_Cs_5.75_In_10_Au at 100K. This led to the assumption of a wiggling of the [Au@In_10_]^9–^ units. Subsequently, anharmonic refinement was applied for different indium atoms (see SI Section 7) for compound (**1**). The anharmonic refinement of the third order was carried out for In4, In5, In6, and In7, whereas for Rb1, Rb2, and Rb4, anharmonic refinement of the fourth order was applied. At 123 K as well as at 100 K, Kuhs' rule is not fulfilled for In4 and In5 (for all of these atoms, a higher resolution would be required).^85^ Also, a negative PDF is found when using anharmonic refinement, but as the values are smaller than 1%, anharmonic refinement is still appropriate. This, in combination with the significant improvement of the crystallographic quality factors of the third- and fourth-order refinement, suggests that anharmonic refinement is reasonable. For compound (**2**), no anharmonic refinement was applied. Here, only two indium positions (In11A/B and In31A/B) were split. In the CIF, the anharmonic refinement is given, whereas in the SI, the quality values for the harmonic as well as the anharmonic refinements are listed.

The site occupancy factors (s.o.f.) of the alkali metal positions Na3, Rb5A, and Rb5b were first refined freely independent. Here, the following s.o.f.’s were found: s.o.f.(Na3) = 0.756(6), s.o.f.(Rb5A) = 0.73(4), and s.o.f.(Rb5B) = 0.240(3). Thus, their occupancies were fixed to the values: s.o.f.(Na3/Rb5A) = 0.75 and s.o.f.(Rb5B) = 0.25, which led to the reported stoichiometry.

Crystallographic data for the compounds have been deposited in the Cambridge Crystallographic Data Center, CCDC, 12 Union Road, Cambridge CB21EZ, UK. Copies of the data can be obtained free of charge under the depository numbers 2475287, 2475288, and 2490369, respectively. (Fax: +44–1223–336–033, E-Mail: deposit@ccdc.cam.ac.uk, http://www.ccdc.cam.ac.uk).

### 2.4 Powder Diffraction Studies

Powder diffraction samples were prepared in sealed capillaries (Ø 0.3 mm, WJM-Glas-Müller GmbH, Berlin, Germany). The data collection was carried out on a STOE Stadi P diffractometer (STOE, Darmstadt, Germany) (monochromatic MoKα1 radiation, λ = 0.70926 Å) equipped with a Dectris Mythen 1 K detector. For visualization and indexation, the software WinXPOW^86^ as well as JANA2006 ^87^ was used.

### 2.5 Solvation Experiments in Liquid Ammonia

In the glovebox, the compounds were weighed into a Schlenk flask, which had been baked out three times before. After that, liquid ammonia was condensed at 195 K on the products using the Schlenk technique. For evaporation, the Schlenk technique was also used again at 195 K. The residue was taken out of the glovebox and pestled in a mortar.

### 2.6 SEM–EDS Measurement

For the SEM/EDS measurement, the crystals were selected and prepared in a glovebox. The measurement was performed on a Zeiss EVO MA15 (Carl Zeiss Microscopy Deutschland GmbH, Oberkochen) using the software SmartSEM Version 6.05 with an accelerating voltage of 25 kV. A Bruker Quantax 200-Z3 xFlash (Bruker Corporation, Billerica, USA) was used as the X-ray detector with the software Bruker Esprit 2.1.2 for EDS measurements.

### 2.7 ^23^Na Solid-State NMR Measurement

NMR measurements for both compounds were performed on an Infinityplus spectrometer system (Agilent) operated at 7 T, equipped with a Chemagnetics–Varian 6 mm pencil cross-polarization magic angle spinning (CPMAS) probe. Spectra were recorded using a 90° pulse of 5.0 μs and a relaxation delay of 1 s. The spectra were indirectly referenced to NaCl (1 M in H_2_O). The NMR parameters of the experimental ^23^Na spectrum were extracted using the WSolids1 simulation software. (K. Eichele, WSolids1. version 1.20.20 2013, Universität Tübingen.)

### 2.8 DFT Calculations

Theoretical calculations of Na_3_Rb_6_In_10_Au were performed on a fully ordered model with reduced symmetry. The structural details of this model can be found in the SI in Chapter 11. The program FPLO21 ^88–91^ was used, which is based on the full-potential non-orthogonal local orbital minimum-basis within the generalized gradient approximation (GGA) for a full-relativistic mode. The exchange-correlation was assumed in the form proposed by Perdew, Burke, and Ernzerhof (PBE).^92^ For the calculation of the density of states (DOS) and the band structure, a modular grid for the reciprocal space of 216 k-points was sufficient. As a convergence criterion, a change of the total energy (Δ*E*_tot_ ≤ 10^–6^ Hartree) was applied. All calculations were carried out without and with spin–orbit coupling (SOC), while no severe difference was observed. For the visualization of the DOS, the program Origin2022 (Version 9.9.0.225) was used.^93^

## 3 Results and Discussion

### 3.1 Crystal Structures of Na_3_Rb_6_In_10_Au (**1**) and Na_3.25_Cs_5.75_In_10_Au (**2**)

#### 3.1.1 Anionic Substructure [Au@In_10_]^9–^

Depending on the alkali metal, two different structure types in two different space groups are observed. Na_3_Rb_6_In_10_Au crystallizes in the monoclinic space group *C*2/*m* (No. 12) (crystallographic details see in SI Chapter 7). According to the *Zintl*-*Klemm*-*Busmann* concept, the alkali metals donate their valence electron, and therefore the compound can be formulated as (Na^+^)_3_(Rb^+^)_6_[Au@In_10_]^9–^.^94–101^ In order to justify this formal concept, the endohedral cluster needs to be described as being built from an innocent Au^+^ and a [In_10_]^10–^ cluster. For similarly shaped, valence-isoelectronic [Ni@In_10_]^10–^, the distortion was reported in literature as driven by the need to destabilize one of the occupied orbitals of the *closo* [In_10_]^12–^ cage.^102^ This description is in line with the *Zintl*-*Klemm*-*Busmann* concept,^94–101^ so formally, [Au@In_10_]^9–^ can also be referred to as a *Zintl*-type cluster. It needs to be emphasized that the *Zintl–Klemm-Busmann* concept in this case is used as a formalism and is not capable of reflecting detailed electronic situations. The anionic entities consist of seven crystallographically independent indium positions (see Figure 1). As it is supposed that the cluster wiggles, an anharmonic refinement was carried out for four indium atoms of the cluster. Atoms sharing the same site but having different local environments also have different local potential energy surfaces. This is assumed to be the reason for this wiggling. Information about the anharmonic refinement can be found in the Experimental Section and in the SI, Chapter 7. In the asymmetric unit, seven crystallographically independent indium positions (Wyckoff sites 4*i*, 8*j*) and one crystallographically independent gold position (Wyckoff sites 4*i*) can be found, which form one [Au@In_10_]^9–^ cluster by mirror symmetry operation. The heavier compound Na_3.25_Cs_5.75_In_10_Au, however, crystallizes in the orthorhombic space group *Pna*2_1_ (No. 33) (crystallographic details see in SI, Chapter 8). In the asymmetric unit, four crystallographically independent [Au@In_10_]^9–^ clusters are present, which leads to 40 crystallographically independent indium positions. Here, no anharmonic refinement had to be used, but a disorder in two [Au@In_10_]^9–^ clusters could be resolved (for more information, see SI, Chapter 8). The shape of all these endohedral [Au@In_10_]^9–^ clusters is comparable to the one in the K_10_In_10_ *M* (*M* = Ni, Pd, Pt) compound,^68^ where two crystallographically distinct indium clusters with different symmetries are described. The here reported [Au@In_10_]^9–^ clusters in (**1**) and (**2**) fit the 50 valence electron count of the above-mentioned [*M*@In_10_]^*x*−^.

**Figure 1:**
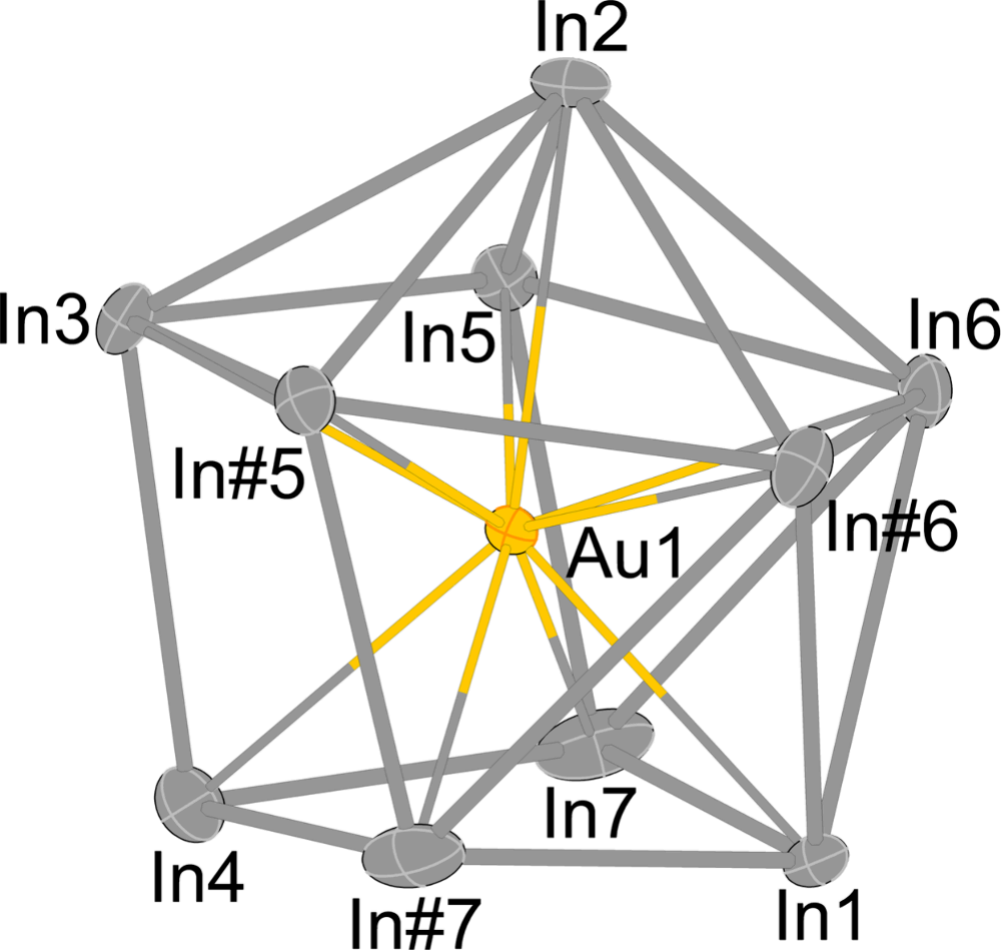
Anionic [Au@In_10_]^9–^ cluster. The only symmetry element is a mirror plane, which goes through In1, In2, In3, and In4, and the endohedral gold atom Au1. In#5 (*x*, 1 – *y*, *z*), In#6 (*x*, 1 – *y*, *z*), and In#7 (*x*, 1 – *y*, *z*) are all generated by this crystallographic mirror plane.

In Na_3_Rb_6_In_10_Au, the [Au@In_10_]^9–^ clusters are located on a mirror plane, which runs through In1, In2, In3, In4, and the coinage metal in the center of the cluster. The endohedral cluster exhibits crystallographic *C*_*s*_ symmetry (see Figure 1), as is known from the one in K_10_In_10_ *M* (*M* = Ni, Pd, and Pt).

The In–In distances in the [Au@In_10_]^9–^ clusters of Na_3_Rb_6_In_10_Au range between 2.99 and 3.46 Å and therefore are comparable with In–In distances reported in literature, where In–In distances between 2.83 and 3.48 Å are observed.^41,43,68,103–105^ The Au–In distances reach from 2.77 up to 2.89 Å, which are also reported in literature (d­(In–Au) = 2.74–2.93 Å).^6,11,12,106,107^

The [Au@In_10_]^9–^ clusters in Na_3_Rb_6_In_10_Au adopt a distorted hexagonal close-packed (hcp) arrangement. Consequently, each cluster is surrounded by 12 neighboring clusters, located at the vertices of a distorted anticuboctahedron (see SI Chapter 7). The same arrangement is reported for the ternary K_10_In_10_ *M* (*M* = Ni, Pd, Pt) compounds.^68^

The heavier compound Na_3.25_Cs_5.75_In_10_Au includes four crystallographically independent [Au@In_10_]^9–^ clusters in the asymmetric unit, each consisting of ten crystallographically independent indium atoms (see SI Chapter 8). The In–In distances in these four endohedral clusters range between 2.98 and 3.49 Å and therefore are also in the expected range as reported in the literature.^41,43,68,103,105^ The same is true for the In–Au distances, which range here from 2.79 to 2.89 Å.^6,11,12,106,107^ This shows that the size of the endohedral [Au@In_10_]^9–^ clusters hardly changes with the heavy alkali metal used. The three-dimensional arrangement of the clusters for (**2**) differs, as no densest packing of the clusters is observed. One [Au@In_10_]^9–^ cluster is surrounded by 14 cluster units. This leads to a total coordination number of 14 (see SI Chapter 8).

#### 3.1.2 Crystal Structure of Na_3_Rb_6_In_10_Au

There are nine crystallographically independent alkali metal sites in the unit cell of Na_3_Rb_6_In_10_Au. They show a special kind of disorder, which is discussed in detail later. The three crystallographically independent sodium positions (Wyckoff positions 2*c*, 4*e,* and 8*j*) show In–Na distances between 3.15 and 3.61 Å, which are comparable to the literature-reported data.^31,42,43^ The six heavier rubidium atoms exhibit longer In–Rb distances, which range from 3.67 to 4.52 Å. Thus, they are also comparable with the reported literature data. For example, in Na_26_ *A*_3_In_48_, the In–Rb distances range from 3.94 to 4.13 Å,^33^ and in Na_7_RbIn_4_, which exhibits isolated [In_4_]^8–^ tetrahedra, the corresponding distances reach from 3.90 to 4.39 Å.^42^ A detailed description of the coordination environments of all alkali metals can be found in the SI in Chapter 7.

Due to their small size, the sodium atoms exhibit small coordination numbers as well as smaller Na–In and Na–Rb distances, respectively, compared to the heavier alkali metals. The significant difference in size of the sodium atoms and the larger alkali metals results in fully ordered alkali metal positions as mixed sites are not favored, and no phase width is observed. This behavior is already known since *Goldschmidt*’s rules^108^ and also reported for example, in Na_4_ *A*_6_Tl_13_ (*A* = K, Rb, Cs)^105^ and Na_3_K_8_Tl_13_.^109^

As a special feature, in the unit cell of (**1**), a disorder is present, which includes split positions of alkali metal atoms (Na3, Rb5A, and Rb5B). The site occupancy factor (s.o.f.) of Rb5A (8*j*) and also Na3 (8*j*) refines to a s.o.f. = 0.75, whereas Rb5B (8*j*) exhibits a s.o.f. of 0.25 (see SI Chapter 7.3). The Na3–Rb5B distance (2.650(3) Å) would be too close; therefore, Rb5B and Na3 cannot be present at the same time. The Rb5A-Na3 distance of 3.486(3) Å is comparable with other Na–Rb distances in the literature.^42,43,33^ The local disorder of the alkali metals does not result in long-range ordering, as no superlattice reflections are observed in the diffraction pattern. The difference electron density was also checked using JANA2020.^110^ Here, it can be seen clearly that the position of Rb5 cannot be described by only one atom and that the two positions also cannot have the same atomic displacement parameter (ADP) (see Figure 2).

**Figure 2:**
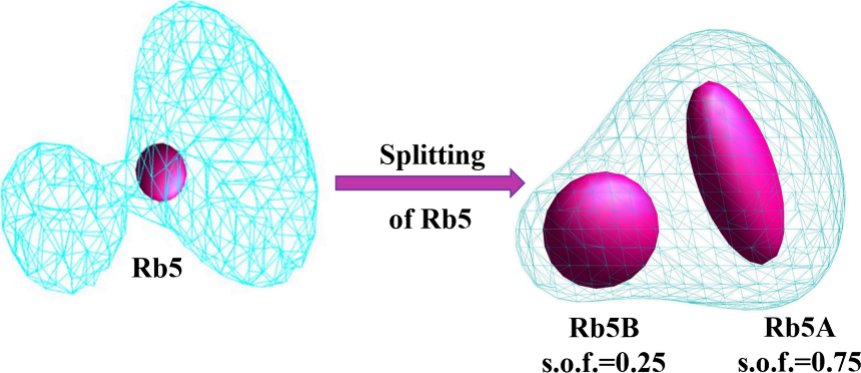
Difference electron density generated in JANA2020 is depicted without Rb5 overlaid with the refined atoms Rb5A and Rb5B, respectively. It demonstrates that the position cannot be described by one atom and that the two close positions cannot have the same ADP.

This disorder leads to the correct stoichiometry of Na_3_Rb_6_In_10_Au. The alkali metal proportion was also experimentally verified as samples deviating from the stoichiometry of Na_3_Rb_6_In_10_Au yielded additional unreacted alkali metal (see SI Chapter 5) next to the compound Na_3_Rb_6_In_10_Au. Therefore, it seems that the alkali metal ratio is fixed and cannot be changed as the clusters need the stated composition of [Au@In_10_]^9–^. The composition was also proven by SEM-EDS measurements (see SI Chapter 9). Altogether, two surroundings of different coordination numbers (CN) are observed for the [Au@In_10_]^9–^ clusters (see Figure 3).

**Figure 3:**
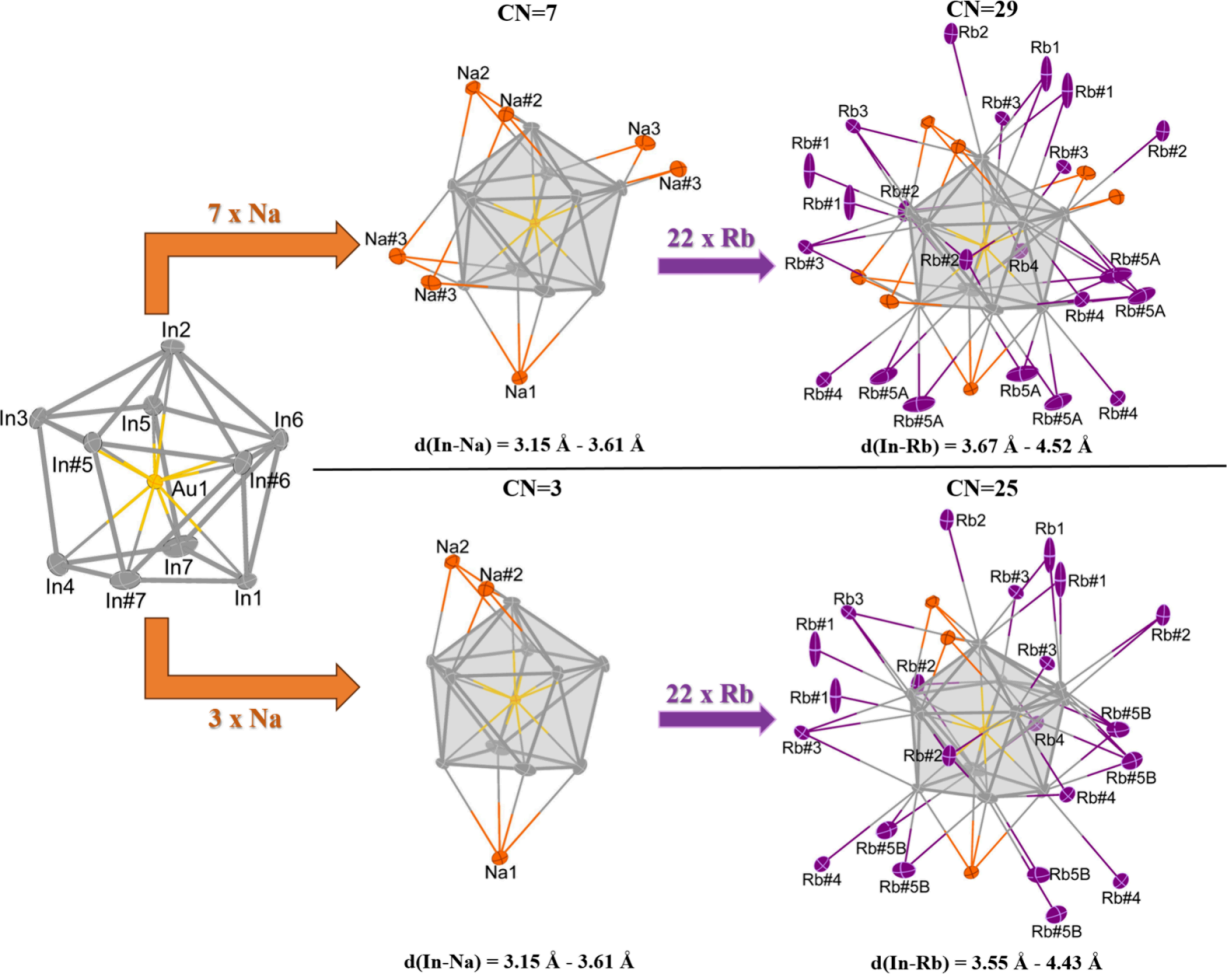
Alkali metal coordination around the endohedral [Au@In_10_]^9–^ cluster. In the upper part, the coordination environment is shown when Rb5A and therefore Na1, Na2, and Na3 are also present. In the lower part, the coordination environment is shown when Rb5B and therefore only Na1 and Na2 are present. Thus, different coordination numbers around the endohedral [Au@In_10_]^9–^ cluster are observed depending on whether Rb5A or Rb5B is considered. Atoms labeled with # are symmetry-generated and therefore are at equivalent positions. The following symmetry operations are used to generate these atom positions: Na2 (3/2 – *x*, 3/2 – *y*, 2 – *z*), Na3 (1 – *x*, +*y*, 1 – *z*), Rb1 (−1/2 + *x*, −1/2 + *y*, −1/2 + *z*), Rb2 (*x*, −1 + *y*, *z*), Rb3 (1 – *x*, +*y*, 2 – *z*), Rb4 (1 – *x*, +*y*, 1 – *z*), Rb5A (1/2 + *x*, 3/2 – *y*, +*z*), and Rb5B (1/2 + *x*, 3/2 – *y*, +*z*).

Whereas the number of rubidium atoms around the endohedral [Au@In_10_]^9–^ cluster always stays the same, the number of sodium atoms changes depending on the rubidium atom present. Rb5A and Na3 are always present at the same time, as discussed above. As there are four Na3 sites generated by symmetry around one endohedral [Au@In_10_]^9–^ cluster, the CN of the indium cluster is higher (CN = 29) when Rb5A and Na3 are present. If Rb5B is considered, only Na1 and Na2 but no Na3 reside in the coordination sphere of the cluster, and therefore the CN is reduced by 4 (CN = 25) (see Figure 3).

#### 3.1.3 Crystal Structure of Na_3.25_Cs_5.75_In_10_Au

In Na_3.25_Cs_5.75_In_10_Au, the crystal system as well as the space group change compared to the lighter Na_3_Rb_6_In_10_Au. In the asymmetric unit, there are 13 crystallographically independent sodium positions (Wyckoff site 4*a*) and 28 cesium positions (Wyckoff site 4*a*). The total number of cesium atoms deviates from 28, as ten positions are not fully occupied but pairwise complementary, yielding a total number of 23 cesium atoms. Further, 45 indium positions (Wyckoff site 4*a*) are observed, of which ten show a disorder, which can be correlated with the cesium atoms and therefore means a concerted whole-structure disorder (see SI Chapter 8). Altogether, the overall composition of nine alkali metals for one [Au@In_10_]^9–^ cluster stays the same, proving the 9-fold negative charge of this new endohedral cluster.

The usage of different alkali metals and especially different alkali metal ratios can lead to a change in the unit cell, as the example of Cs_1–*x*_Rb_*x*_Tl shows.^59^ Here, two new monoclinic compounds were obtained (Cs_0.82_Rb_0.18_Tl and Cs_0.58_Rb_0.42_Tl) depending on the cesium content in the structure. As expected, the more cesium that is present, the larger the received unit cell is, while the anionic entity [Tl_6_]^6–^ remains the same.

Taking a look at the surroundings of the four crystallographically independent [Au@In_10_]^9–^ clusters shows that three of them exhibit a CN of 28, whereas the fourth one shows a CN of 29 alkali metals in its near surroundings (see SI Chapter 8). Along the crystallographic *b-*direction, a disorder of ten cesium positions is observed but without influencing the total coordination number of the [Au@In_10_]^9–^ clusters in (**2**).

#### 3.1.4 Dissolution Experiments in Liquid Ammonia

Liquid ammonia is a frequently used solvent for highly charged *Zintl* anions^111–113^ and has been successfully employed for the synthesis of new *Zintl*-type clusters.^100,114–117^ While group 14–16 *Zintl* solution chemistry in this solvent is well-known, the reactivity of group 13 *Zintl* phases is rarely reported. While often oxidation to the elements (indium or thallium) is observed,^57,58^ only a few examples are known that show reactivity.^43,118^ Therefore, the solution behavior of (**1**) and (**2**) in liquid ammonia was investigated (see SI Chapter 6). First, the compounds were stored in the solvent for 1 week. After evaporation of the liquid ammonia, no reaction was observed (see SI Chapter 6.1). In contrast, when Na_3_Rb_6_In_10_Au is stored in liquid ammonia for 10 weeks, after evaporation of the solvent, In_2_Au and elemental indium could be identified in the powder diffraction pattern (see SI Chapter 6.2). The heavier compound Na_3.25_Cs_5.75_In_10_Au also shows a reaction in liquid ammonia after 10 weeks. Here, mainly the compound itself could be identified next to some In_2_Au (see SI Chapter 6.3). As Na_3.25_Cs_5.75_In_10_Au still is preserved after 10 weeks, it can be stated that the compound Na_3.25_Cs_5.75_In_10_Au is less reactive than Na_3_Rb_6_In_10_Au, which also might be attributed to a different solubility in this solvent.

#### 3.1.5 ^23^Na Solid-State NMR Spectroscopy

^23^Na static NMR spectra of (**1**) and (**2**) both display a single broad signal in the range of −200 to 400 ppm (see SI Chapter 10). The spectra can be simulated using the following spectroscopic parameters: δ_iso_ ≈ 165 ppm (**1**)/150 ppm (**2**), C_Q_ ≈ 4.95 MHz (**1**)/4.7 MHz (**2**), and η ≈ 0 (**1**, **2**). The slightly different coupling constants may be attributed to the presence of different vicinal heavy alkali metals (rubidium or cesium). The environments of all sodium centers are similar and approximately axially symmetric (see SI, Chapter 7), which is reflected in the axial symmetry of the signals. The quadrupolar coupling constant is large, and the asymmetry parameter of the electric field gradient tensor is small. The available MAS rate (5 kHz) is insufficient to simplify the spectrum when the quadrupolar coupling constant is so large. Sample (**1**) cannot be spun, while sample (**2**) can be spun. However, in the latter case, the spinning rate gradually decreases and can be restored by increasing the drive pressure. In both cases, a possible reason is the degradation of the substances with the formation of a metallic material caused by pressure from centrifugal forces. A slower reaction time for (**2**) was also observed in the dissolution experiments in liquid ammonia, where metallic products formed in both cases.

#### 3.1.6 Theoretical Calculations

Due to the rubidium split position, a fully ordered model with lower symmetry (SG *P* $\overset{-}{1}$ ) of Na_3_Rb_6_In_10_Au was created (see SI, Chapter 11.4) for the electronic structure calculations. A small band gap of 0.5 eV clearly depicts the salt-like behavior of the compound (see Figure 4). The anionic nature of the indium cluster is shown by the largest contribution to the highest occupied states. The Au–In interactions are represented by a high DOS from −3 to −4 eV, at −5.5 eV, and between −6.5 and −7 eV (see SI, Chapter 11). The conduction band consists of a larger amount of sodium and rubidium states, as expected, for the cations. The calculated band structures for the ordered cell can be found in the Supporting Information, Chapter 11.

**Figure 4:**
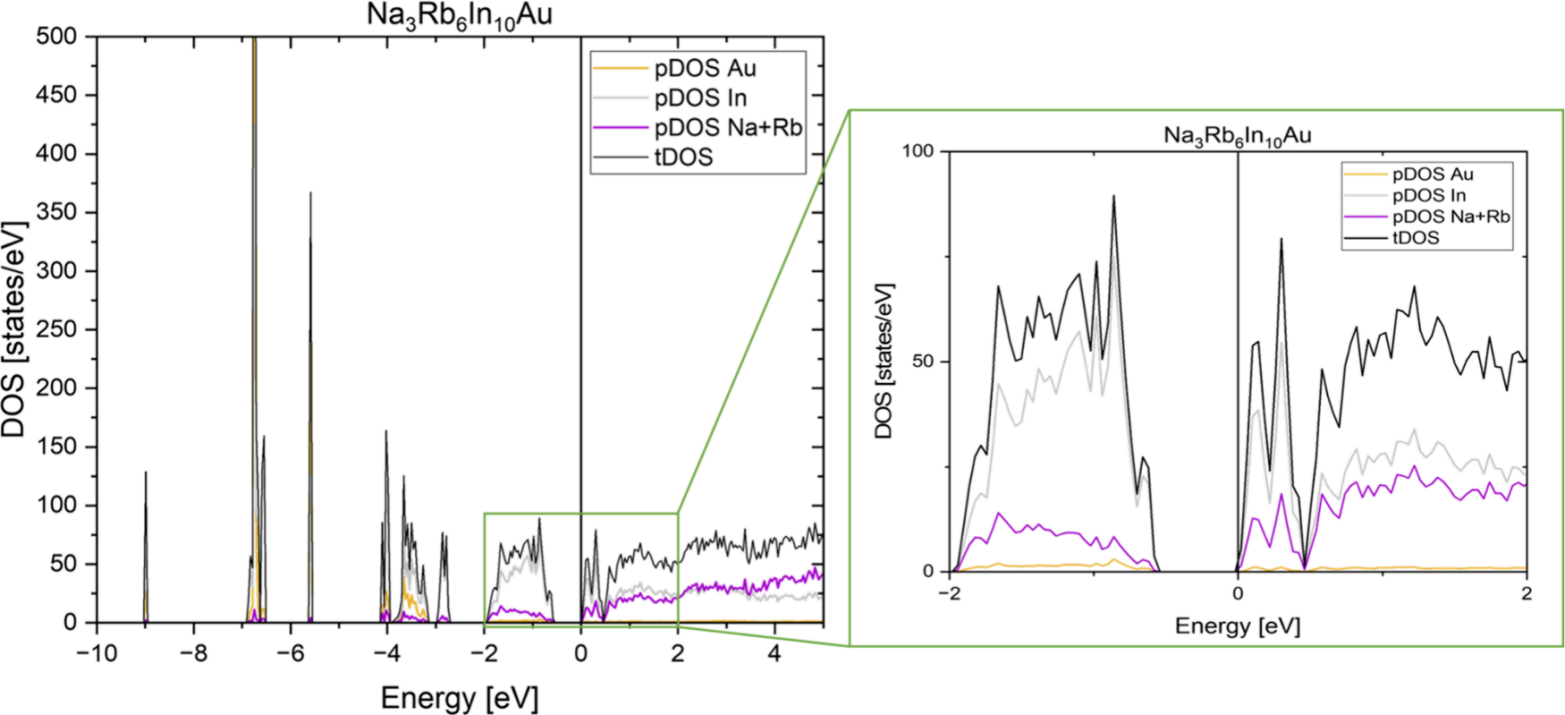
Density of states of the compound Na_3_Rb_6_In_10_Au with the total DOS (tDOS, black) and the partial DOS (pDOS) of indium (gray), the alkali metals Na and Rb (purple), and gold (yellow).

## 4 Conclusions

For a long period of time, it was assumed that small quantities of coinage metals do not have any influence on the intermetallic structure of alkali metal indides. With the here-reported structures Na_3_Rb_6_In_10_Au and Na_3.25_Cs_5.75_In_10_Au, however, it could be shown that the incorporation of gold can indeed stabilize naked [Au@In_10_]^9–^ clusters in the solid state. Depending on the alkali metal, different space groups are observed, whereas the anionic entity remains the same. The outstanding feature of Na_3_Rb_6_In_10_Au is the arrangement of the alkali metals refined by split positions, which is a necessity to realize the exact chemical composition. This alkali metal composition was independently verified by the preparation of different stoichiometric approaches and SEM/EDS measurements, which always resulted in the reported composition. Na_3.25_Cs_5.75_In_10_Au, however, crystallizes in a different space group, and its better crystallinity is considered to be the reason for a different dissolution behavior in liquid ammonia. Here, for Na_3_Rb_6_In_10_Au, a complete reaction to elemental indium and In_2_Au was observed after 10 weeks, whereas for Na_3.25_Cs_5.75_In_10_Au, unreacted educt remained, next to In_2_Au. This might be quite interesting in terms of a new pathway for the preparation of new materials, as In_2_Au recently received much attention due to its technological importance.^119–125^ In general, these two compounds showed again that the different alkali metals are not only innocent counterions but play a crucial role in the formation of new *Zintl-*type clusters.

## Supplementary Material



## Notes

Experimental and DFT calculation details, crystallographic data, additional structure description, powder diffraction patterns, SEM/EDS and ^23^Na solid-state NMR (PDF)
