# Supplementary material for: Endohedral [Au@In10]9– Cluster: Synthesis and Characterization of Na3+x A 6–x In10Au (x = 0, 0.25; A = Rb, Cs)
Source: Inorg Chem. 2026 Jan 30;65(6):3343–51. doi: 10.1021/acs.inorgchem.5c04619 (PMC12914637; doi:10.1021/acs.inorgchem.5c04619)
Supplement: Supplementary file 1 [file ic5c04619_si_001.pdf]

Supporting Information

Endohedral $[Au@In_{10}]^{9-}$ Cluster: Synthesis and Characterization of $Na_{3+x}A_{6-x}In_{10}Au$ (x=0, 0.25; A=Rb, Cs)

Melissa Janesch[(a)], Florian Pielnhofer[(a)], Michal Dušek[(b)], Ilya G. Shenderovich[(c)], Stefanie Gärtner[(a,c)]*

(a) Institute of Inorganic Chemistry, University of Regensburg, Universitätsstr. 31, 93053 Regensburg, Germany
(b) Institute of Physics of the Czech Academy of Sciences, Na Slovance 2, 182 21 Prague 8, Czech Republic
(c) Central Analytics, University of Regensburg, Universitätsstr. 31, 93053 Regensburg, Germany

*Corresponding author: stefanie.gaertner@ur.de

Content

1. Crystallographic Data of $Na_3Rb_6In_{10}Au$ 3
2. Crystallographic of $Na_{3.25}Cs_{5.75}In_{10}Au$ 4
3. Atomic coordinates 5
3.1 $Na_3Rb_6In_{10}Au$ at 123K 5
3.2 $Na_{3.25}Cs_{5.75}In_{10}Au$ 5
4. Displacement Parameters 7
4.1 $Na_3Rb_6In_{10}Au$ at 123K 7
4.2 $Na_{3.25}Cs_{5.75}In_{10}Au$ 8
5. Powder Diffraction Pattern 10
5.1 Powder Diffraction Pattern of $Na_3Rb_6In_{10}Au$ with normal temperature program 10
5.2 Powder Diffraction Pattern of $Na_3Rb_6In_{10}Au$ after annealing 11
5.3 Powder Diffraction Pattern of $Na_3Rb_6In_{10}Au$ after quenching 11
5.4 Powder Diffraction Pattern of $Na_4Rb_5In_{10}Au$ 12
5.5 Powder Diffraction Pattern of $Na_{3.25}Cs_{5.75}In_{10}Au$ with normal temperature program 13
5.6 Powder Diffraction Pattern of $Na_{3.25}Cs_{5.75}In_{10}Au$ after annealing 13
5.7 Powder Diffraction Pattern of $Na_{3.25}Cs_{5.75}In_{10}Au$ after quenching 14
6. Powder diffraction pattern of the dissolution experiments in liquid ammonia 15
6.1 Powder diffraction pattern of $Na_3Rb_6In_{10}Au$ after evaporation of liquid ammonia after one week ... 15
6.2 Powder diffraction pattern of $Na_3Rb_6In_{10}Au$ after evaporation of liquid ammonia after one month . 15
6.3 Powder diffraction pattern of $Na_{3.25}Cs_{5.75}In_{10}Au$ after evaporation of liquid ammonia after one month 17
7. Details of the Crystal Structure of $Na_3Rb_6In_{10}Au$ 17
7.1 Unit cell and packing sequence of the anionic entities 17
7.2 Coordination environment of the alkali metals 18
7.3 Splitting of Rb5 in $Na_3Rb_6In_{10}Au$ 20
7.4 X-Ray Structure solution 21
8. Details of the Crystal Structure of $Na_{3.25}Cs_{5.75}In_{10}Au$ 24
8.1 Unit cell and packing sequence of the anionic entities 24
8.2 The four crystallographic independent $[Au@In_{10}]^{9-}$ clusters in $Na_{3.25}Cs_{5.75}In_{10}Au$ 25
9. SEM/EDS spectra 28
10. ^{23}Na-Solid-State-NMR spectroscopy 29
11. Theoretical calculations 30
11.1 In-Au Interactions in the Density of states 30
11.2 Scalar relativistic band structure plot of $Na_3Rb_6In_{10}Au$ 31
11.3 Full relativistic band structure plot of $Na_3Rb_6In_{10}Au$ 31
11.4 CIF file of the fully ordered mode for $Na_3Rb_6In_{10}Au$ 32
References 34

1. Crystallographic Data of $Na_3Rb_6In_{10}Au$

Table S 1. *Crystallographic Data for $Na_3Rb_6In_{10}Au$ at 123 K and at 100 K.*

**Empirical formula**	$Na_3Rb_6In_{10}Au$	$Na_3Rb_6In_{10}Au$
**CCDC number**	2475288	2475287
**Formula weight**	1926.92	
**Temperature/K**	123	100
**Crystal system**	monoclinic	
**Space group**	*C*2/*m*	
***a*/Å**	16.3056(1)	16.2918(1)
***b*/Å**	11.3364(1)	11.3242(1)
***c*/Å**	16.3778(1)	16.3680(1)
**β/°**	101.271(1)	101.300(1)
**Volume/Å^3**	2969.00(4)	2961.22(4)
Z	4	
**ρ_{calc}/g/cm^3**	4.311	4.322
**μ/mm^{-1}**	22.323	22.381
**F(000)**	3243.8	3243.8
**Crystal size/mm^3**	0.15 × 0.04 × 0.03	0.07 × 0.03 × 0.03
**Radiation**	Mo Kα (λ = 0.71073)	
**2Θ range for data collection/°**	4.4 to 75.6	4.4 to 78.38
**Index ranges**	$-29 \le h \le 29$, $-20 \le k \le 20$, $-29 \le l \le 26$	$-27 \le h \le 28$, $-20 \le k \le 19$, $-28 \le l \le 28$
**Reflections collected**	156636	102966
**Independent reflections**	8272	8874
**Data/restraints/parameters**	8272/300/180	8874/0/193
**R_{int}**	0.0368	0.0258
**Goodness-of-fit on F^2**	1.034	1.038
**Final R indexes [I>=2σ (I)]**	R_1 = 0.0277, wR_2 = 0.0651	R_1 = 0.0263, wR_2 = 0.0526
**Final R indexes [all data]**	R_1 = 0.0351, wR_2 = 0.0677	R_1 = 0.0302, wR_2 = 0.0535
**Largest diff. peak/hole / e Å^{-3}**	4.61/-3.59	3.16/-3.61

2. Crystallographic of $Na_{3.25}Cs_{5.75}In_{10}Au$

Table S 2. *Crystallographic Data for $Na_{3.25}Cs_{5.75}In_{10}Au$ at 100 K.*

**Empirical formula**	$Na_{3.25}Cs_{5.75}In_{10}Au$
**CCDC number**	2490369
**Formula weight**	2184.12
**Temperature/K**	100
**Crystal system**	orthorhombic
**Space group**	$Pna2_1$
***a*/Å**	32.6446(3)
***b*/Å**	16.25586(14)
***c*/Å**	23.2167(2)
**Volume/Å^3**	12320.31(19)
Z	16
**ρ_{calc}g/cm^3**	4.710
**μ/mm^{-1}**	18.806
**F(000)**	14736
**Crystal size/mm^3**	0.13 × 0.1 × 0.07
**Radiation**	Mo Kα (λ = 0.71073)
**2Θ range for data collection/°**	4.982 to 72.638
**Index ranges**	$-50 \le h \le 54$, $-27 \le k \le 15$, $-37 \le l \le 38$
**Reflections collected**	152961
**Independent reflections**	52756
**Data/restraints/parameters**	52756/1/771
**R$_{int}$**	0.0323
**Goodness-of-fit on F^2**	1.082
**Final R indexes [I>=2σ (I)]**	R_1 = 0.0339, wR_2 = 0.0678
**Final R indexes [all data]**	R_1 = 0.0379, wR_2 = 0.0690
**Largest diff. peak/hole / e Å^{-3}**	2.19/-3.43
**Flack parameter**	0.477(3)

3. Atomic coordinates

3.1 $Na_3Rb_6In_{10}Au$ at 123K

Table S 3. *Fractal atomic coordinates and equivalent isotropic displacement parameters for $Na_3Rb_6In_{10}Au$.*

**Atom (Wyckoff site)**	***x***	***y***	***z***	***U*(eq)/Å²**
Au1 (4*i*)	0.689092(9)	0.5	0.754362(9)	0.00847(3)
In1 (4*i*)	0.512417(19)	0.5	0.70340(2)	0.01475(5)
In2(4*i*)	0.770263(14)	0.72008(2)	0.798225(14)	0.01486(4)
In3 (4*i*)	0.78638(2)	0.5	0.92275(2)	0.01551(6)
In4 (4*i*)	0.60911(3)	0.63617(5)	0.86369(4)	0.01746(5)
In5A (8*j*)	0.85998(5)	0.5	0.74898(6)	0.02045(7)
In6 (8*j*)	0.62164(4)	0.30053(6)	0.65849(4)	0.03010(8)
In7 (8*j*)	0.71552(7)	0.5	0.59138(6)	0.04141(14)
Rb1 (4*g*)	0.61895(9)	1	0.81615(10)	0.0338(3)
Rb2 (4*i*)	0.5	0.82866(6)	1	0.0562(3)
Rb3 (4*i*)	0.39017(3)	0.5	0.90731(4)	0.02247(9)
Rb4 (4*i*)	0.73367(11)	0.5	0.35625(10)	0.0378(4)
Rb5A (8*j*)	0.38260(7)	0.78811(8)	0.55774(5)	0.0607(3)
Rb5B (8*j*)	0.40230(15)	0.77948(19)	0.60673(14)	0.0407(4)
Na1 (2*c*)	0.5	0.5	0.5	0.0225(6)
Na2 (4*e*)	0.75	0.75	1	0.0204(4)
Na2 (8*j*)	0.45460(15)	0.7522(2)	0.76957(15)	0.0272(4)

3.2 $Na_{3.25}Cs_{5.75}In_{10}Au$

Table S 4. *Fractal atomic coordinates and equivalent isotropic displacement parameters for $Na_{3.25}Cs_{5.75}In_{10}Au$. All atoms reside on Wyckoff site 4a (1).*

**Atom**	***x***	***y***	***z***	***U*(eq)/Å²**
Au1	0.87622(2)	0.74129(2)	0.42387(2)	0.00920(5)
Au2	0.88201(2)	1.24417(2)	0.67100(2)	0.00913(4)
Au3	0.62171(2)	0.24707(2)	0.41357(2)	0.00937(5)
Au4	0.62510(2)	0.75050(2)	0.66347(2)	0.00934(5)
Cs1	0.77064(2)	0.14619(5)	0.35473(4)	0.03107(15)
Cs2	0.7746(3)	0.1844(8)	0.3735(6)	0.03107(15)
Cs3	0.81170(2)	0.34555(4)	0.44243(4)	0.02748(14)
Cs4	0.7795(3)	0.3840(6)	0.5086(6)	0.031(3)
Cs5	0.72092(3)	0.42377(5)	0.55932(4)	0.0408(2)
Cs6	0.6813(2)	0.3543(4)	0.6612(5)	0.0188(19)
Cs7	0.78737(2)	0.58113(4)	0.67189(4)	0.02120(11)
Cs8	0.7770(3)	0.6002(7)	0.6758(6)	0.02120(11)
Cs9	0.7296(3)	1.1201(6)	0.6651(8)	0.036(3)
Cs10	0.72732(2)	1.16897(5)	0.65525(4)	0.03439(17)
Cs11	0.73337(2)	0.95913(5)	0.76952(4)	0.03787(17)
Cs12	0.82125(2)	0.84233(3)	0.67338(3)	0.01963(10)
Cs13	0.76046(2)	1.00704(4)	0.51440(3)	0.02472(12)
Cs14	1.00137(2)	0.99338(3)	0.83153(3)	0.01618(10)
Cs15	0.95189(2)	1.40482(3)	0.41808(3)	0.01803(10)

Cs16	0.91058(2)	1.15839(3)	0.42663(3)	0.01806(10)
Cs17	0.99989(2)	0.99977(3)	0.50494(3)	0.01580(10)
Cs18	0.90560(2)	0.66794(3)	0.17508(3)	0.01886(10)
Cs19	0.72353(2)	0.60061(3)	0.40918(3)	0.02457(12)
Cs20	0.58407(2)	0.66129(3)	0.41898(3)	0.01614(9)
Cs21	0.55376(2)	0.40860(3)	0.67042(3)	0.01875(9)
Cs22	0.50350(2)	0.00489(3)	0.58313(3)	0.01494(9)
Cs23	0.50014(2)	-0.00732(3)	0.25465(3)	0.01451(9)
Cs24	0.67448(2)	-0.15552(3)	0.42259(3)	0.01736(9)
Cs25	0.54287(2)	0.41180(3)	0.16851(3)	0.01487(8)
Cs26	0.41809(2)	0.83791(3)	0.67035(3)	0.01504(8)
Cs27	0.54415(2)	0.91581(3)	0.41832(2)	0.01439(9)
Cs28	0.76944(2)	0.88280(4)	0.28008(4)	0.03276(15)
Na1	1.00041(13)	0.7480(2)	0.5434(2)	0.0165(6)
Na2	1.00249(12)	1.2449(2)	0.79700(18)	0.0164(7)
Na3	0.50451(12)	0.7539(2)	0.79408(18)	0.0174(7)
Na4	0.62012(14)	0.5023(2)	0.5437(2)	0.0245(9)
Na5	0.62349(12)	0.9999(2)	0.80161(19)	0.0207(8)
Na6	0.87509(12)	1.0039(2)	0.54962(18)	0.0179(7)
Na7	0.87288(13)	1.4965(2)	0.55026(18)	0.0202(8)
Na8	0.62795(13)	0.5017(2)	0.7943(2)	0.0212(8)
Na9	0.86901(12)	1.0003(2)	0.79672(18)	0.0183(7)
Na10	0.74904(13)	0.7451(2)	0.55078(19)	0.0243(8)
Na11	0.63143(12)	0.0083(2)	0.54621(18)	0.0187(7)
Na12	0.87064(12)	1.4986(2)	0.79360(19)	0.0191(7)
Na13	0.50523(12)	0.2527(2)	0.54563(18)	0.0163(7)
In1	0.86862(2)	0.91592(3)	0.42141(3)	0.01736(11)
In2	0.79630(2)	0.79528(4)	0.43122(3)	0.01822(11)
In3	0.82823(2)	0.68881(3)	0.32582(3)	0.01534(10)
In4	0.89693(2)	0.81264(3)	0.31686(3)	0.01385(10)
In5	0.95940(2)	0.80291(3)	0.42110(3)	0.01277(9)
In6	0.90124(2)	0.81860(4)	0.52766(3)	0.01475(10)
In7	0.83737(2)	0.68699(4)	0.52719(3)	0.01591(11)
In8	0.84781(2)	0.57520(3)	0.42426(3)	0.01370(10)
In9	0.92921(2)	0.63841(3)	0.35633(3)	0.01151(9)
In10	0.93076(2)	0.63622(3)	0.48874(3)	0.01192(10)
In11	0.81232(2)	1.34047(4)	0.65505(4)	0.02120(14)
In12	0.82543(2)	1.21610(4)	0.76224(3)	0.02246(13)
In13	0.84750(2)	1.08421(3)	0.67443(3)	0.01509(10)
In14	0.83468(2)	1.19624(4)	0.57269(3)	0.01606(11)
In15	0.90714(2)	1.31217(3)	0.56261(3)	0.01242(10)
In16	0.89502(2)	1.41696(3)	0.67262(3)	0.01488(10)
In17	0.90459(2)	1.30913(3)	0.77990(3)	0.01354(10)
In18	0.92978(2)	1.13386(3)	0.73924(3)	0.01212(10)
In19	0.93294(2)	1.13514(3)	0.60534(3)	0.01131(9)
In20	0.96862(2)	1.28839(3)	0.67109(3)	0.01155(8)

In21	0.68731(3)	0.21729(5)	0.48991(4)	0.0375(2)
In22	0.60858(2)	0.30477(4)	0.52746(3)	0.02098(13)
In23	0.61069(2)	0.41850(3)	0.42089(3)	0.01630(11)
In24	0.69150(2)	0.34271(4)	0.38553(4)	0.02885(16)
In25	0.65291(2)	0.08370(3)	0.41619(3)	0.01448(10)
In26	0.57472(2)	0.13982(3)	0.48481(3)	0.01261(10)
In27	0.53585(2)	0.29401(3)	0.41968(3)	0.01250(9)
In28	0.59193(2)	0.31860(3)	0.30810(3)	0.01252(10)
In29	0.66154(2)	0.19572(4)	0.31063(3)	0.01639(11)
In30	0.56851(2)	0.13728(3)	0.35029(3)	0.01172(10)
In31	0.62757(3)	0.79567(4)	0.78140(3)	0.02021(13)
In32	0.61200(2)	0.92130(4)	0.67886(3)	0.01358(12)
In33	0.68379(2)	0.86007(4)	0.61158(3)	0.01720(12)
In34	0.70438(2)	0.74501(4)	0.70810(4)	0.02423(15)
In35	0.57898(2)	0.64947(3)	0.73754(3)	0.01474(10)
In36	0.54046(2)	0.80086(3)	0.67151(3)	0.01264(9)
In37	0.59419(2)	0.82010(3)	0.55763(3)	0.01245(10)
In38	0.66105(2)	0.68172(3)	0.56201(3)	0.01412(10)
In39	0.65852(2)	0.58747(3)	0.67639(3)	0.01685(11)
In40	0.57037(2)	0.64032(3)	0.60406(3)	0.01203(10)
In41	0.8052(3)	1.3185(7)	0.6745(7)	0.02120(14)
In42	0.6057(4)	0.8092(7)	0.7778(5)	0.02021(13)
In43	0.6240(4)	0.9189(8)	0.6743(7)	0.01358(12)
In44	0.6979(3)	0.8241(6)	0.6450(5)	0.01720(12)
In45	0.6779(3)	0.7031(7)	0.7580(6)	0.02423(15)

4. Displacement Parameters

4.1 $Na_3Rb_6In_{10}Au$ at 123K

Table S 5. *Anisotropic Displacement Parameters (Å^{2}x10^3) for $Na_3Rb_6In_{10}Au$. The anisotropic displacement factor exponent takes the form: $-2\pi^2[h^2a^{*2}U_{11}+2hka^*b^*U_{12}+\ldots]$.*

**Atom**	U_{11}	U_{22}	U_{33}	U_{12}	U_{13}	U_{23}
Au1	0.00882(6)	0.00891(6)	0.00743(5)	-0.000000	0.00100(4)	-0.000000
In1	0.00953(11)	0.01822(13)	0.01476(12)	-0.000000	-0.00190(9)	-0.000000
In2	0.01639(9)	0.01042(8)	0.01830(9)	-0.00408(7)	0.00474(7)	-0.00104(7)
In3	0.01913(13)	0.01243(12)	0.01152(11)	-0.000000	-0.00548(10)	-0.000000
In4	0.01302(9)	0.01759(10)	0.02334(11)	-0.00091(7)	0.00744(8)	-0.00940(8)
In5	0.01089(12)	0.01406(13)	0.0381(2)	-0.000000	0.00891(12)	-0.000000
In6	0.02945(14)	0.01954(12)	0.03406(16)	0.00537(10)	-0.01166(12)	-0.01515(11)
In7	0.0275(2)	0.0848(4)	0.01465(16)	-0.000000	0.01092(15)	-0.000000
Rb1	0.0314(7)	0.0294(7)	0.0450(9)	-0.000000	0.0186(6)	-0.000000
Rb2	0.0564(5)	0.0145(2)	0.1191(8)	-0.000000	0.0698(5)	-0.000000
Rb3	0.0191(2)	0.0192(2)	0.0274(2)	-0.000000	0.00043(17)	-0.000000
Rb4	0.0350(9)	0.0542(13)	0.0250(7)	-0.000000	0.0079(6)	-0.000000
Rb5A	0.0979(7)	0.0507(4)	0.0262(3)	0.0403(4)	-0.0061(3)	-0.0116(3)
Rb5B	0.0518(10)	0.0346(8)	0.0330(8)	0.0232(7)	0.0020(6)	-0.0049(6)

Na1	0.0230(13)	0.0273(14)	0.0168(12)	-0.000000	0.0027(9)	-0.000000
Na2	0.0241(9)	0.0189(8)	0.0187(8)	-0.0030(6)	0.0051(6)	-0.0060(5)
Na3	0.0277(9)	0.0243(8)	0.0273(8)	0.0144(6)	-0.0004(5)	-0.0017(5)

4.2 $Na_{3.25}Cs_{5.75}In_{10}Au$

Table S 6. *Anisotropic Displacement Parameters (Å^{2}x10^3) for $Na_{3.25}Cs_{5.75}In_{10}Au$. The anisotropic displacement factor exponent takes the form: $-2\pi^2[h^2a^{*2}U_{11}+2hka^*b^*U_{12}+...]$.*

**Atom**	U_{11}	U_{22}	U_{33}	U_{12}	U_{13}	U_{23}
Au1	0.00959(11)	0.00816(9)	0.00984(13)	0.00011(8)	0.00033(10)	-0.00031(10)
Au2	0.01027(10)	0.00771(9)	0.00940(11)	0.00038(7)	-0.00012(11)	-0.00038(10)
Au3	0.01048(12)	0.00766(9)	0.00998(13)	-0.00017(8)	-0.00037(10)	0.00006(9)
Au4	0.00949(10)	0.00858(9)	0.00995(13)	0.00043(8)	-0.00008(9)	-0.00065(9)
Cs1	0.0219(3)	0.0333(3)	0.0380(4)	0.0015(3)	0.0003(3)	-0.0011(3)
Cs2	0.0219(3)	0.0333(3)	0.0380(4)	0.0015(3)	0.0003(3)	-0.0011(3)
Cs3	0.0253(3)	0.0194(2)	0.0378(4)	-0.0013(2)	-0.0082(3)	0.0046(3)
Cs4	0.030(5)	0.021(4)	0.043(7)	-0.005(4)	-0.024(5)	0.015(4)
Cs5	0.0332(4)	0.0435(4)	0.0456(5)	0.0091(3)	-0.0175(4)	-0.0167(4)
Cs6	0.007(3)	0.007(3)	0.042(6)	0.001(2)	-0.005(3)	-0.007(3)
Cs7	0.0215(3)	0.0175(2)	0.0245(3)	0.00182(18)	0.0029(3)	-0.0004(3)
Cs8	0.0215(3)	0.0175(2)	0.0245(3)	0.00182(18)	0.0029(3)	-0.0004(3)
Cs9	0.018(4)	0.012(3)	0.077(10)	-0.003(3)	0.010(6)	-0.003(5)
Cs10	0.0216(3)	0.0444(4)	0.0372(4)	-0.0086(3)	0.0043(3)	-0.0091(3)
Cs11	0.0277(3)	0.0431(4)	0.0428(4)	0.0008(3)	-0.0013(3)	-0.0170(3)
Cs12	0.0208(2)	0.01543(18)	0.0226(3)	-0.00047(16)	-0.0010(2)	-0.0007(2)
Cs13	0.0194(3)	0.0236(2)	0.0311(3)	-0.0018(2)	-0.0009(2)	-0.0028(2)
Cs14	0.0193(2)	0.0136(2)	0.0156(2)	0.00160(17)	0.00029(18)	0.00103(18)
Cs15	0.0193(2)	0.01511(19)	0.0197(3)	0.00154(16)	0.0033(2)	0.0026(2)
Cs16	0.0198(2)	0.0175(2)	0.0169(2)	-0.00153(17)	0.0004(2)	-0.00077(19)
Cs17	0.0184(2)	0.0140(2)	0.0151(2)	0.00285(18)	0.00107(18)	-0.00004(17)
Cs18	0.0215(2)	0.01552(18)	0.0195(3)	-0.00297(16)	0.0004(2)	-0.0005(2)
Cs19	0.0198(3)	0.0205(2)	0.0333(3)	0.00055(19)	0.0022(2)	-0.0046(2)
Cs20	0.0177(2)	0.01386(18)	0.0169(2)	0.00008(15)	-0.0007(2)	0.00007(19)
Cs21	0.0251(2)	0.01466(18)	0.0165(2)	-0.00090(16)	-0.0002(2)	0.0006(2)
Cs22	0.0167(2)	0.0134(2)	0.0148(2)	-0.00097(16)	0.00083(18)	-0.00027(17)
Cs23	0.0158(2)	0.01332(19)	0.0144(2)	-0.00133(16)	0.00053(18)	-0.00022(17)
Cs24	0.0165(2)	0.01560(18)	0.0200(2)	0.00098(16)	-0.0012(2)	-0.00019(19)
Cs25	0.01655(19)	0.01355(17)	0.0145(2)	-0.00002(14)	-0.0008(2)	0.00063(19)
Cs26	0.0162(2)	0.01400(17)	0.0150(2)	0.00033(14)	0.0002(2)	0.00058(19)
Cs27	0.0157(2)	0.01312(17)	0.0143(2)	0.00026(15)	-0.00005(18)	0.00055(18)
Cs28	0.0364(4)	0.0269(3)	0.0349(4)	-0.0021(2)	-0.0155(3)	0.0047(3)
Na1	0.0135(14)	0.0179(14)	0.0183(16)	-0.0011(12)	-0.0036(12)	0.0000(13)
Na2	0.0152(17)	0.0171(15)	0.0169(17)	-0.0016(12)	-0.0022(13)	0.0005(13)
Na3	0.0168(17)	0.0171(15)	0.0182(18)	0.0012(12)	0.0022(14)	-0.0004(13)
Na4	0.033(2)	0.0150(16)	0.026(2)	0.0010(15)	-0.0036(18)	-0.0062(16)
Na5	0.0235(19)	0.0201(16)	0.018(2)	0.0024(14)	-0.0024(15)	-0.0081(15)

Na6	0.0185(17)	0.0148(15)	0.0205(19)	-0.0001(12)	0.0001(15)	-0.0057(14)
Na7	0.030(2)	0.0141(15)	0.0170(18)	0.0037(14)	-0.0019(16)	0.0042(14)
Na8	0.0221(18)	0.0184(16)	0.023(2)	-0.0009(14)	-0.0027(15)	0.0074(14)
Na9	0.0211(18)	0.0180(15)	0.0159(18)	0.0015(13)	-0.0005(14)	0.0031(13)
Na10	0.0162(17)	0.0316(19)	0.025(2)	0.0025(14)	0.0011(16)	0.0004(17)
Na11	0.0224(19)	0.0175(16)	0.0160(18)	0.0033(13)	-0.0017(14)	0.0005(14)
Na12	0.0218(18)	0.0171(15)	0.0186(18)	0.0009(14)	0.0027(15)	-0.0028(13)
Na13	0.0154(18)	0.0176(15)	0.0158(16)	0.0015(12)	0.0025(14)	-0.0005(13)
In1	0.0302(3)	0.00854(19)	0.0133(3)	0.00078(19)	0.0013(2)	-0.0002(2)
In2	0.0119(2)	0.0237(3)	0.0191(3)	0.00570(19)	0.0011(2)	0.0007(2)
In3	0.0165(2)	0.0153(2)	0.0142(3)	-0.00005(19)	-0.0044(2)	-0.0017(2)
In4	0.0170(2)	0.0130(2)	0.0115(2)	0.00190(18)	0.0016(2)	0.00221(19)
In5	0.0119(2)	0.0138(2)	0.0126(2)	-0.00373(16)	0.0007(2)	-0.0006(2)
In6	0.0185(3)	0.0138(2)	0.0119(2)	0.00119(19)	-0.0017(2)	-0.0032(2)
In7	0.0149(3)	0.0180(2)	0.0148(3)	0.00248(19)	0.0046(2)	0.0033(2)
In8	0.0159(2)	0.01004(19)	0.0152(3)	-0.00254(17)	-0.0011(2)	0.0008(2)
In9	0.0116(2)	0.0099(2)	0.0130(2)	0.00047(17)	0.00188(19)	-0.00198(18)
In10	0.0114(2)	0.0106(2)	0.0137(2)	0.00093(17)	-0.0014(2)	0.00099(19)
In11	0.0129(3)	0.0171(3)	0.0336(4)	0.0053(2)	-0.0016(3)	0.0006(3)
In12	0.0196(3)	0.0302(3)	0.0176(3)	-0.0060(2)	0.0079(2)	-0.0043(2)
In13	0.0183(2)	0.01093(19)	0.0161(3)	-0.00413(16)	-0.0021(2)	0.0010(2)
In14	0.0172(3)	0.0174(2)	0.0136(3)	-0.0037(2)	-0.0041(2)	0.0004(2)
In15	0.0157(2)	0.0110(2)	0.0106(2)	0.00083(17)	0.00044(19)	0.00113(18)
In16	0.0234(3)	0.00845(18)	0.0128(2)	0.00012(16)	-0.0005(2)	-0.0004(2)
In17	0.0181(3)	0.0119(2)	0.0106(2)	0.00101(18)	-0.0016(2)	-0.00180(19)
In18	0.0138(2)	0.0097(2)	0.0129(2)	0.00017(17)	-0.00168(19)	0.00153(18)
In19	0.0120(2)	0.0092(2)	0.0128(2)	0.00073(16)	0.00141(19)	-0.00182(18)
In20	0.0111(2)	0.01160(18)	0.0120(2)	-0.00180(15)	-0.0003(2)	-0.0002(2)
In21	0.0380(4)	0.0295(3)	0.0451(5)	0.0147(3)	-0.0309(4)	-0.0151(3)
In22	0.0365(4)	0.0151(2)	0.0114(3)	-0.0082(2)	0.0042(3)	-0.0029(2)
In23	0.0264(3)	0.00831(19)	0.0142(3)	0.00062(18)	0.0010(2)	-0.0008(2)
In24	0.0170(3)	0.0202(3)	0.0494(5)	-0.0080(2)	0.0054(3)	-0.0059(3)
In25	0.0174(2)	0.01018(19)	0.0159(3)	0.00269(17)	0.0010(2)	0.0005(2)
In26	0.0140(2)	0.0100(2)	0.0139(2)	0.00049(17)	0.0019(2)	0.00198(19)
In27	0.0122(2)	0.0133(2)	0.0119(2)	0.00292(16)	0.0005(2)	0.0007(2)
In28	0.0154(2)	0.0111(2)	0.0110(2)	0.00005(17)	-0.00104(19)	0.00172(18)
In29	0.0183(3)	0.0176(2)	0.0132(3)	0.0032(2)	0.0036(2)	-0.0001(2)
In30	0.0124(2)	0.0095(2)	0.0132(3)	-0.00101(17)	-0.00156(19)	-0.00130(18)
In31	0.0341(4)	0.0160(3)	0.0106(3)	-0.0024(3)	-0.0005(3)	-0.0014(2)
In32	0.0168(3)	0.00911(19)	0.0148(3)	0.0017(2)	0.0012(3)	-0.00131(19)
In33	0.0146(3)	0.0138(2)	0.0232(3)	-0.00294(19)	0.0056(2)	-0.0001(2)
In34	0.0157(3)	0.0225(3)	0.0345(4)	0.0022(2)	-0.0113(3)	-0.0015(3)
In35	0.0157(3)	0.0132(2)	0.0154(3)	0.00237(18)	0.0036(2)	0.0040(2)
In36	0.0110(2)	0.01351(19)	0.0135(2)	0.00293(15)	0.0016(2)	0.0009(2)
In37	0.0142(2)	0.0118(2)	0.0113(2)	-0.00086(17)	-0.00033(19)	0.00175(18)
In38	0.0138(2)	0.0144(2)	0.0141(3)	-0.00077(18)	0.0042(2)	-0.0027(2)

In39	0.0187(2)	0.0124(2)	0.0195(3)	0.00516(17)	0.0043(2)	0.0031(2)
In40	0.0112(2)	0.0097(2)	0.0152(2)	-0.00083(16)	-0.00094(19)	-0.00173(19)
In41	0.0129(3)	0.0171(3)	0.0336(4)	0.0053(2)	-0.0016(3)	0.0006(3)
In42	0.0341(4)	0.0160(3)	0.0106(3)	-0.0024(3)	-0.0005(3)	-0.0014(2)
In43	0.0168(3)	0.00911(19)	0.0148(3)	0.0017(2)	0.0012(3)	-0.00131(19)
In44	0.0146(3)	0.0138(2)	0.0232(3)	-0.00294(19)	0.0056(2)	-0.0001(2)
In45	0.0157(3)	0.0225(3)	0.0345(4)	0.0022(2)	-0.0113(3)	-0.0015(3)

5. Powder Diffraction Pattern

5.1 Powder Diffraction Pattern of $Na_3Rb_6In_{10}Au$ with normal temperature program

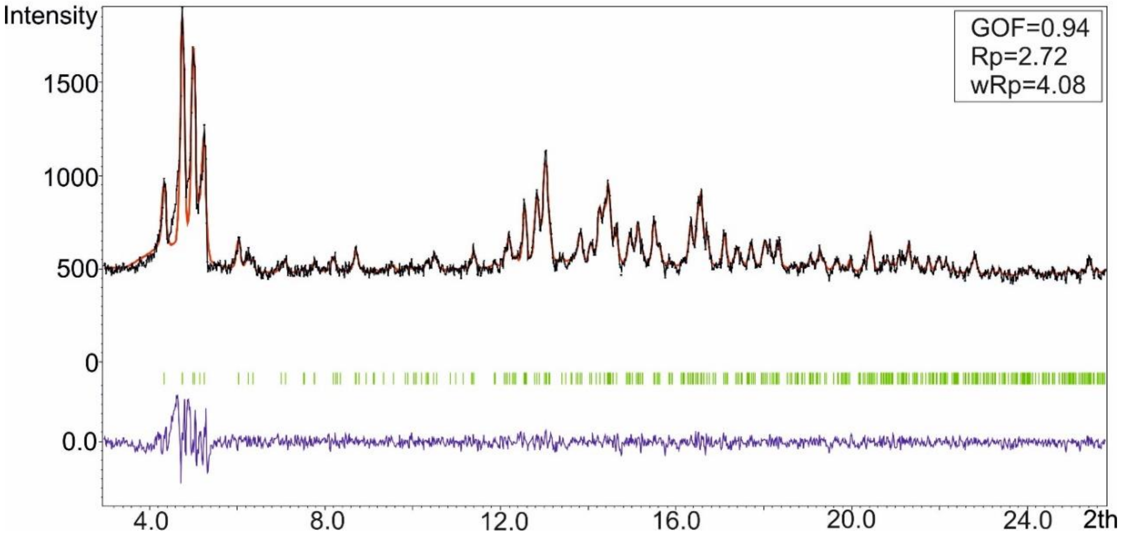

Figure S 1. *Powder diffraction pattern of $Na_3Rb_6In_{10}Au$ synthesized with the following temperature program: Heating from room temperature (r.t.) to 973.15 K with 100 K/h, holding for two days. Then the sample was cooled down to r.t. with 3 K/h. The refinement was carried out using the program JANA2006 with the LeBail algorithm. GOF = 0.94, R_p = 2.72, wR_p = 4.08. The big reflection in the front cannot be indexed properly due to asymmetric broadening.*

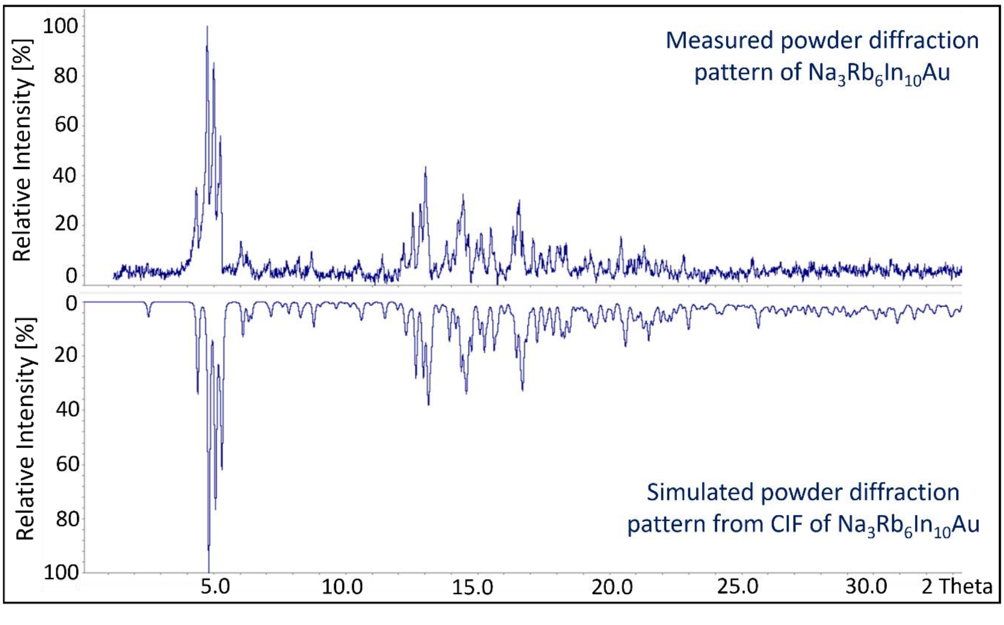

Figure S 2. *Comparison of the measured (above) and the simulated from CIF file of the measured single crystal (down) powder diffraction pattern of $Na_3Rb_6In_{10}Au$. As is can be seen from this pattern the compound is phase pure.*

5.2 Powder Diffraction Pattern of $Na_3Rb_6In_{10}Au$ after annealing

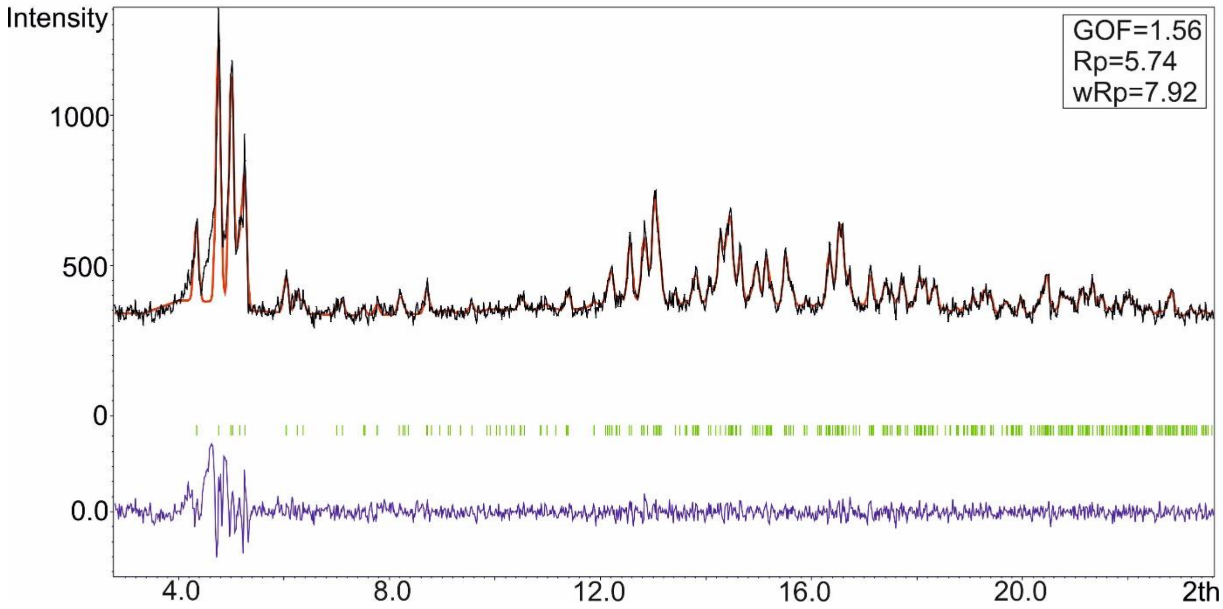

Figure S 3. *Powder diffraction pattern of $Na_3Rb_6In_{10}Au$. The refinement was carried out using the program JANA2006 with the LeBail algorithm. GOF = 1.56, R_p = 5.74, wR_p = 7.92. The big reflection in the front cannot be indexed properly due to asymmetric broadening. The temperature program used for this compound was the following: Heating from r.t. to 973.15 K with 100 K/h, holding for two days. Then the sample was taken out of the furnace, cooled down and annealed for five days at 523.15 K. After that the ampoule was cooled down to r.t. with 3 K/h.*

5.3 Powder Diffraction Pattern of $Na_3Rb_6In_{10}Au$ after quenching

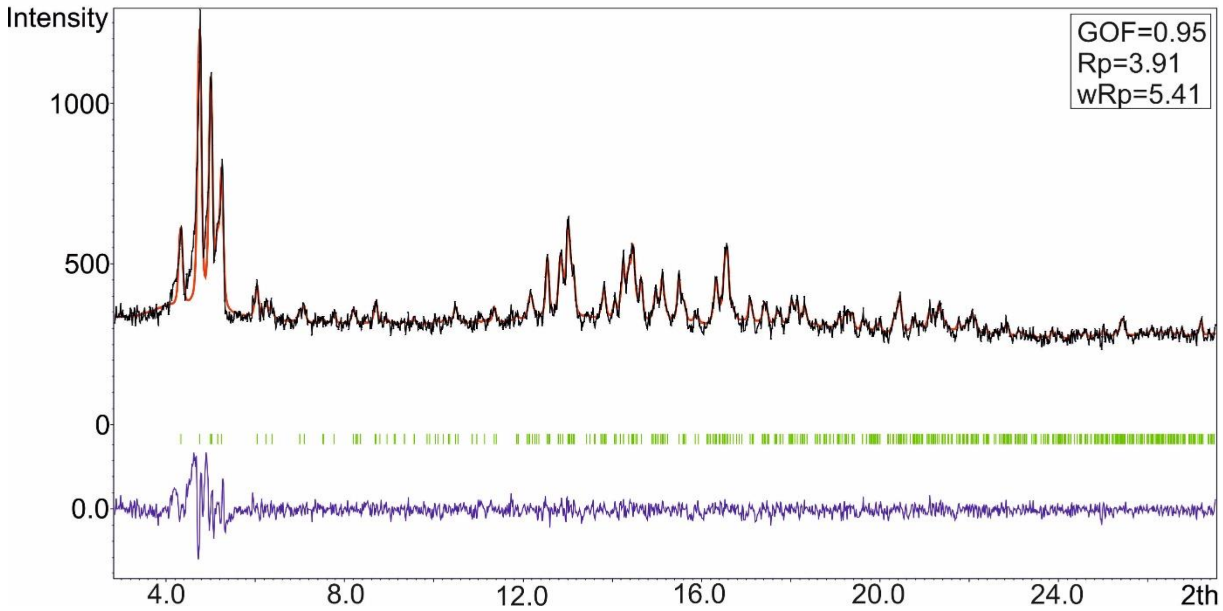

Figure S 4. *Powder diffraction pattern of $Na_3Rb_6In_{10}Au$. The refinement was carried out using the program JANA2006 with the LeBail algorithm. GOF = 0.95, R_p = 3.91, wR_p = 5.41. The big reflection in the front cannot be indexed properly due to asymmetric broadening. The temperature program used for this compound was the following: Heating from r.t. to 973.15 K with 100 K/h, holding for two days. Then the sample was quenched to r.t..*

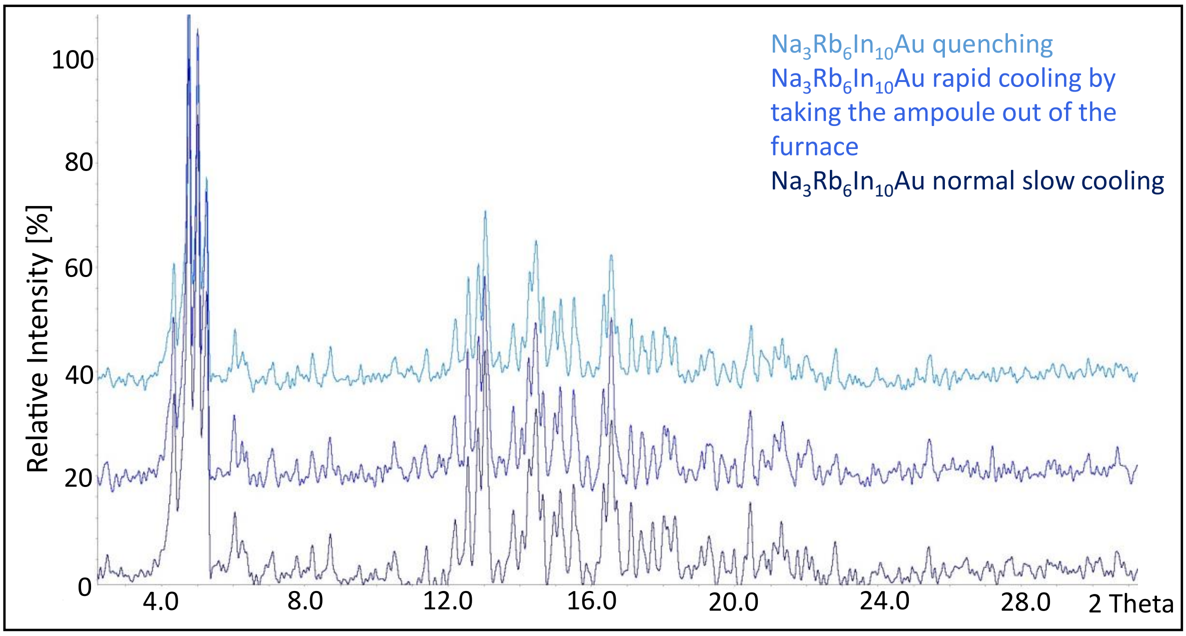

Figure S 5. *The role of the temperature program was investigated for the compound $Na_3Rb_6In_{10}Au$. It seems that the temperature program does not seem to make any difference for the resulting compound, only crystal quality suffers from quenching.*

5.4 Powder Diffraction Pattern of $Na_4Rb_5In_{10}Au$

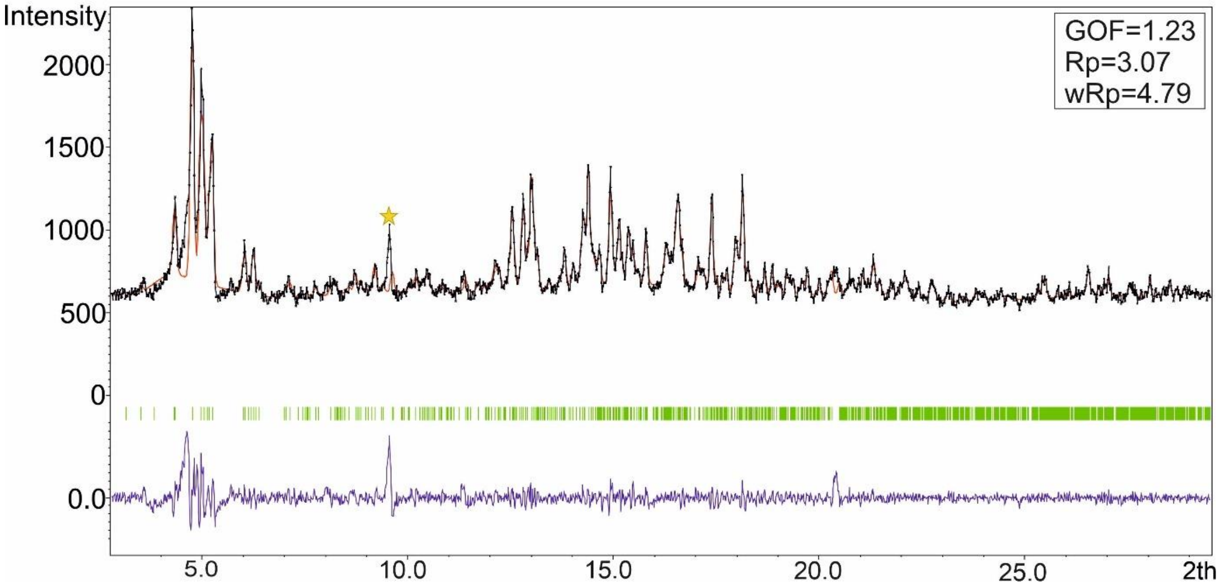

Figure S 6. *Powder Diffraction Pattern of the approach $Na_4Rb_5In_{10}Au$. The refinement was carried out using the program JANA2006 with the LeBail algorithm. GOF = 1.23, R_p = 3.07, wR_p = 4.79. The big reflection in the front cannot be indexed properly due to asymmetric broadening. The main product here is $Na_3Rb_6In_{10}Au$. The one reflection marked with a star could not be assigned to any known compound.*

5.5 Powder Diffraction Pattern of $Na_{3.25}Cs_{5.75}In_{10}Au$ with normal temperature program

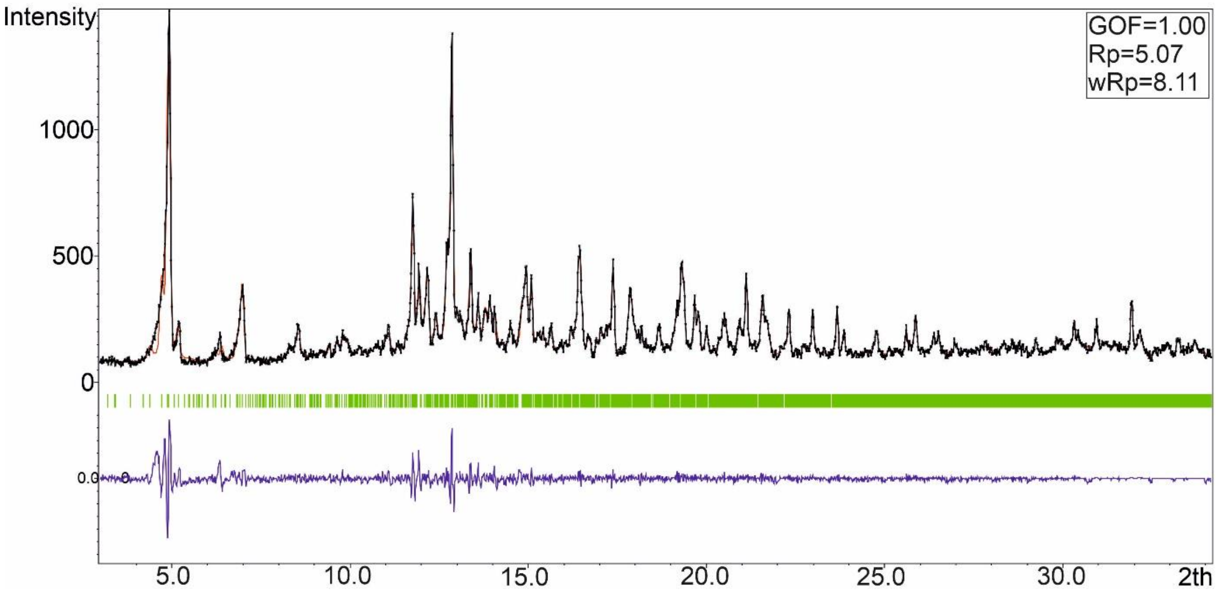

Figure S 7. *Powder diffraction pattern of $Na_{3.25}Cs_{5.75}In_{10}Au$ synthesized with the following temperature program: Heating from room temperature (r.t.) to 973.15 K with 100 K/h, holding for two days. Then the sample was cooled down to r.t. with 3 K/h. The refinement was carried out using the program JANA2006 with the LeBail algorithm. GOF = 1.00, R_p = 5.07, wR_p = 8.11. The big reflection in the front cannot be indexed properly due to asymmetric broadening.*

5.6 Powder Diffraction Pattern of $Na_{3.25}Cs_{5.75}In_{10}Au$ after annealing

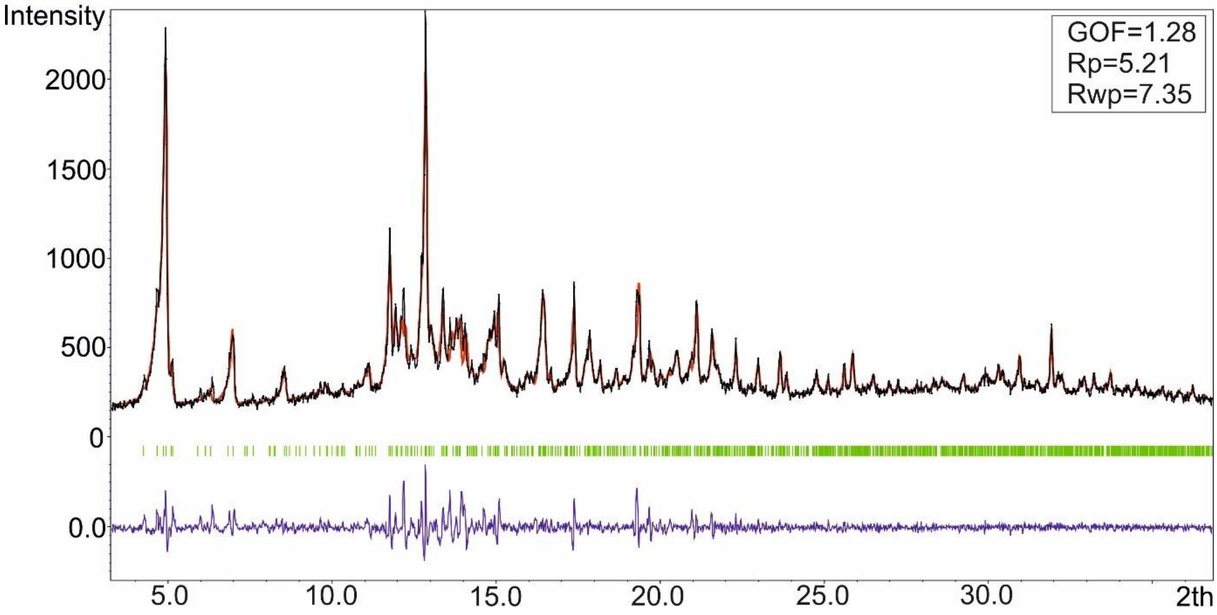

Figure S 8. *Powder diffraction pattern of $Na_{3.25}Cs_{5.75}In_{10}Au$. The refinement was carried out using the program JANA2006 with the LeBail algorithm. GOF = 1.28, R_p = 5.21, wR_p = 7.35. The big reflection in the front cannot be indexed properly due to asymmetric broadening. The temperature program used for this compound was the following: Heating from r.t. to 973.15 K with 100 K/h, holding for two days. Then the sample was taken out of the furnace, cooled down and annealed for five days at 523.15 K. After that the ampoule was cooled down to r.t. with 3 K/h.*

5.7 Powder Diffraction Pattern of $Na_{3.25}Cs_{5.75}In_{10}Au$ after quenching

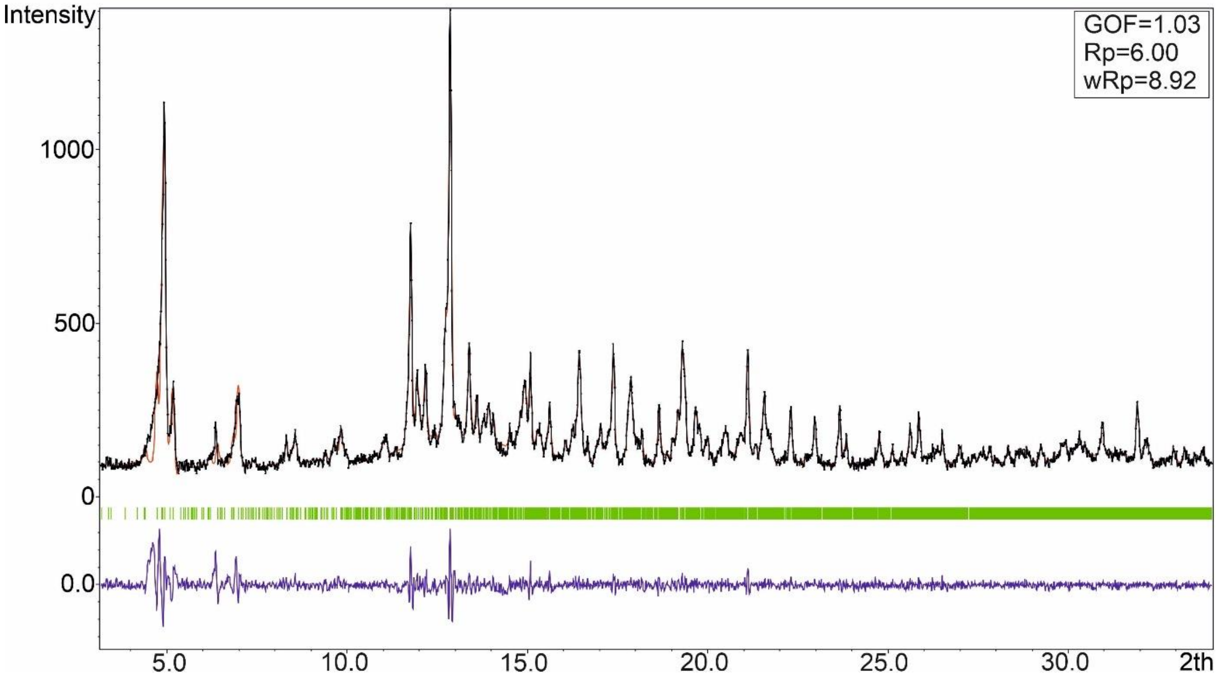

Figure S 9. *Powder diffraction pattern of $Na_{3.25}Cs_{5.75}In_{10}Au$. The refinement was carried out using the program JANA2006 with the LeBail algorithm. GOF = 1.03, R_p = 6.00, wR_p = 8.92. The big reflection in the front cannot be indexed properly due to asymmetric broadening. The temperature program used for this compound was the following: Heating from r.t. to 973.15 K with 100 K/h, holding for two days. Then the sample was quenched to r.t..*

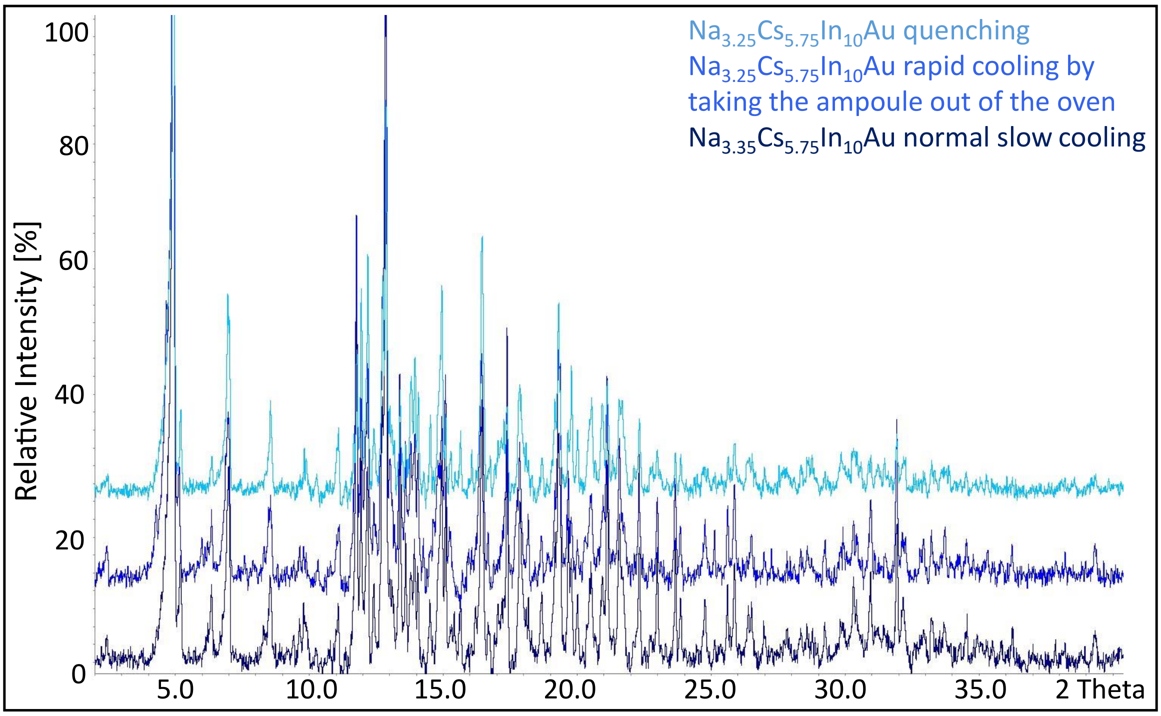

Figure S 10. *The role of the temperature program was investigated for the compound $Na_{3.25}Cs_{5.75}In_{10}Au$. Also here. it seems that the temperature program does not seem to make any difference for the resulting compound, only crystal quality suffers from quenching.*

6. Powder diffraction pattern of the dissolution experiments in liquid ammonia

6.1 Powder diffraction pattern of $Na_3Rb_6In_{10}Au$ after evaporation of liquid ammonia after one week

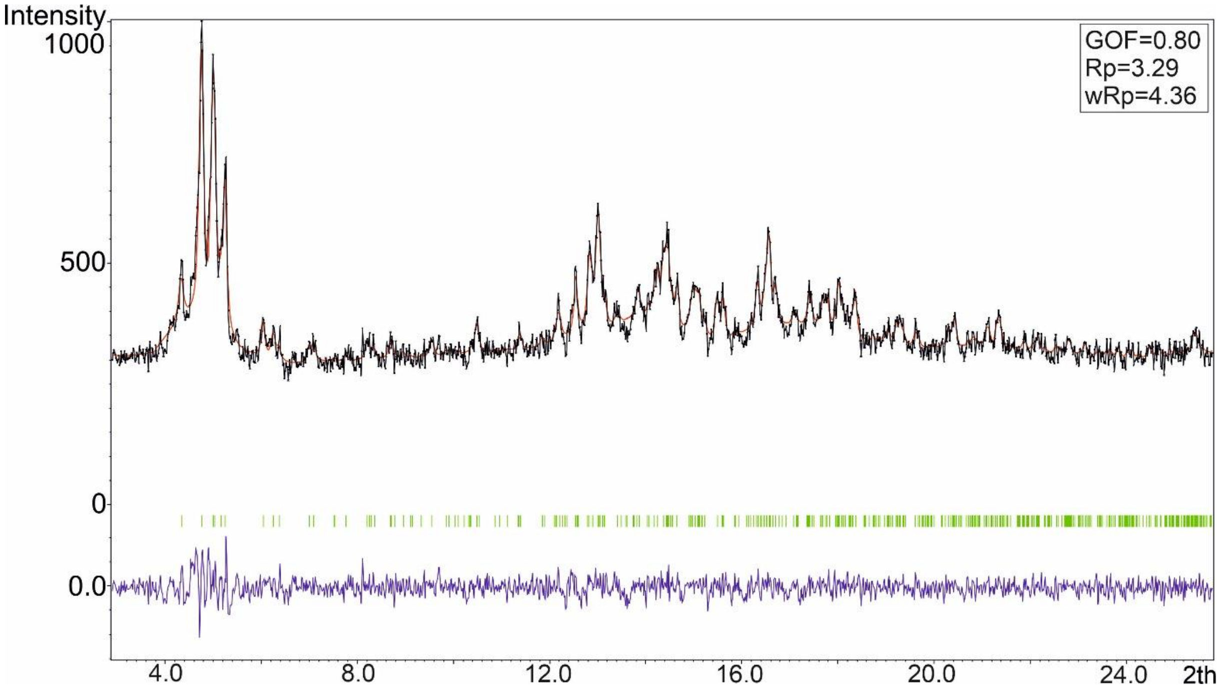

Figure S 11. *Powder diffraction pattern of $Na_3Rb_6In_{10}Au$ after evaporation of liquid ammonia after one week. The refinement was carried out using the program JANA2006 with the LeBail algorithm. GOF = 0.80, R_p = 3.29, wR_p = 4.36. The compound $Na_3Rb_6In_{10}Au$ stayed unreacted and was obtained phase pure.*

6.2 Powder diffraction pattern of $Na_3Rb_6In_{10}Au$ after evaporation of liquid ammonia after one month

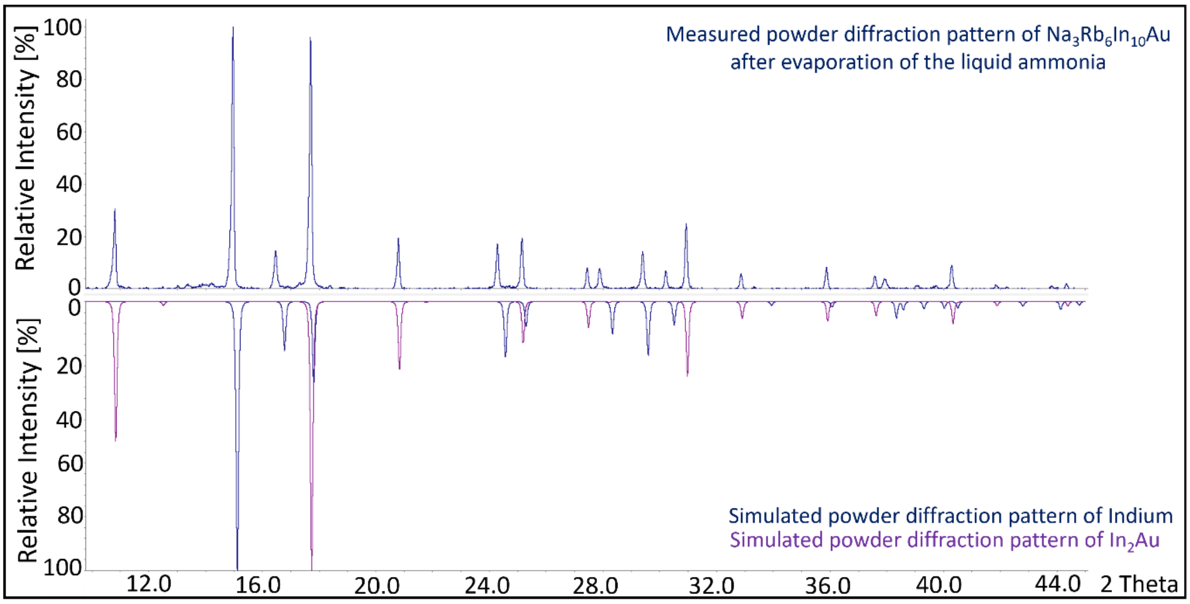

Figure S 12. *Powder diffraction pattern of $Na_3Rb_6In_{10}Au$ after evaporation of liquid ammonia after one month. The compound reacted to elemental indium and In_2Au.*

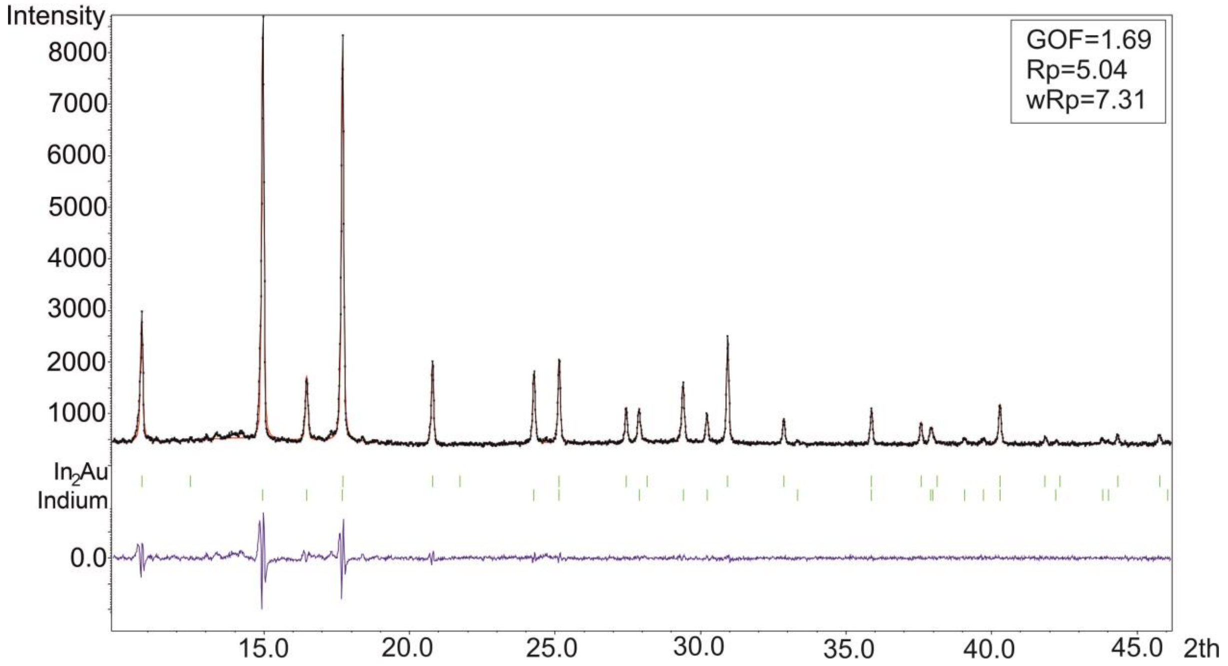

Figure S 13. *Powder diffraction pattern of $Na_3Rb_6In_{10}Au$ after evaporation of liquid ammonia. The refinement was carried out using the program JANA2006 with the LeBail algorithm. GOF = 1.67, R_p = 5.04, wR_p = 7.31. The compound was stored ten weeks in this solvent. There, a reaction to elemental indium and In_2Au took place. It is supposed that the alkali metals reacted to alkali metal amide, which however is not crystalline so it cannot be seen in the powder diffraction pattern.*

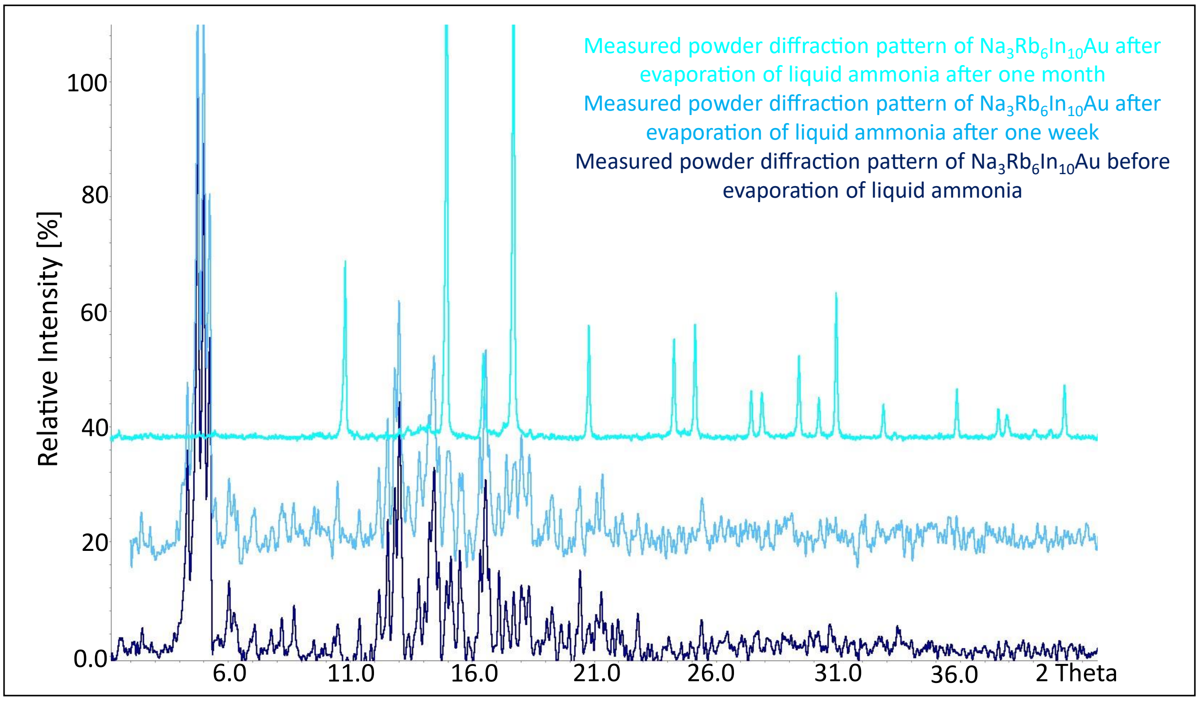

Figure S 14. *Reaction kinetics of the compound $Na_3Rb_6In_{10}Au$ in liquid ammonia. The lowermost powder diffraction pattern shows the compound $Na_3Rb_6In_{10}Au$ before condensation of liquid ammonia. Above the powder diffraction pattern after evaporation of the liquid ammonia after one week is shown and the uppermost powder diffraction pattern depicts the reaction of $Na_3Rb_6In_{10}Au$ after the evaporation of liquid ammonia after one month. As one can see clearly there is a time dependent reaction in liquid ammonia. After one week no reaction occurred but after one month the compound is oxidized to elemental indium as well as Au_2In.*

6.3 Powder diffraction pattern of $Na_{3.25}Cs_{5.75}In_{10}Au$ after evaporation of liquid ammonia after one month

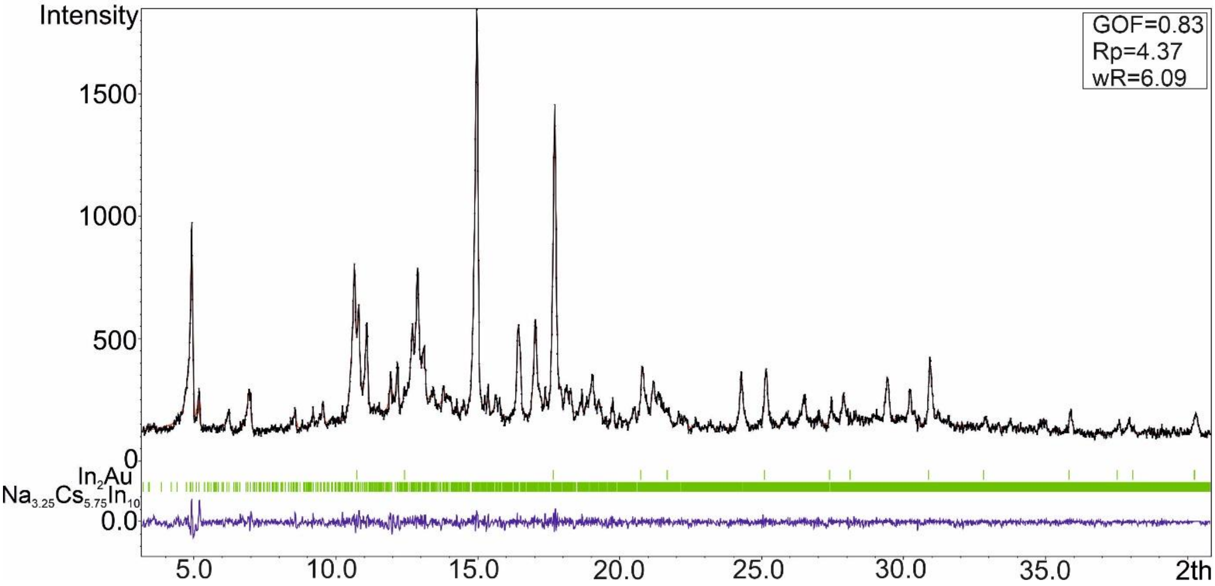

Figure S 15. *Powder diffraction pattern of $Na_{3.25}Cs_{5.75}In_{10}Au$ after evaporation of liquid ammonia. The refinement was carried out using the program JANA2006 with the LeBail algorithm. GOF = 0.83, R_p = 4.37, wR_p = 6.09. The compound was stored ten weeks in this solvent. There, unreacted $Na_{3.25}Cs_{5.75}In_{10}Au$ as well as In_2Au can be identified.*

7. Details of the Crystal Structure of $Na_3Rb_6In_{10}Au$

7.1 Unit cell and packing sequence of the anionic entities

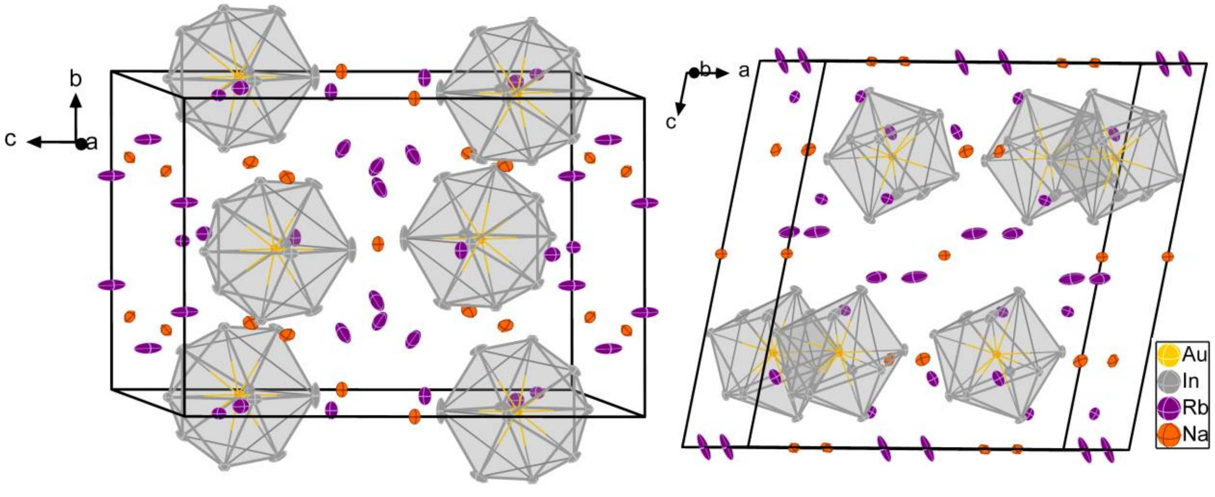

Figure S 16. *Unit cell of the compound $Na_3Rb_6In_{10}Au$ in the crystallographic a- (left) and b- (right) direction in the centrosymmetric space group C2/m.*

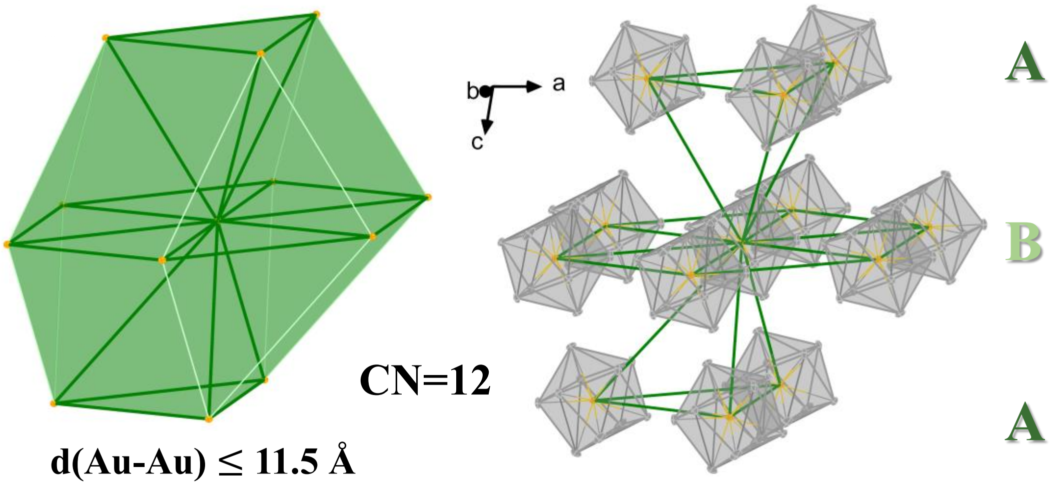

Figure S 17. *Along the crystallographic c-axis a strongly distorted hexagonal AB stacking sequence of the [Au@In$_{10}$]$^{9-}$ clusters is observed in the Na$_3$Rb$_6$In$_{10}$Au structure type as it is known from the hdp. The centers of gravity of the clusters, which correspond to the Au atoms, show distances underneath 11.5 Å.*

7.2 Coordination environment of the alkali metals

As expected, the three crystallographic independent sodium atoms show smaller coordination numbers than the six bigger alkali metals.

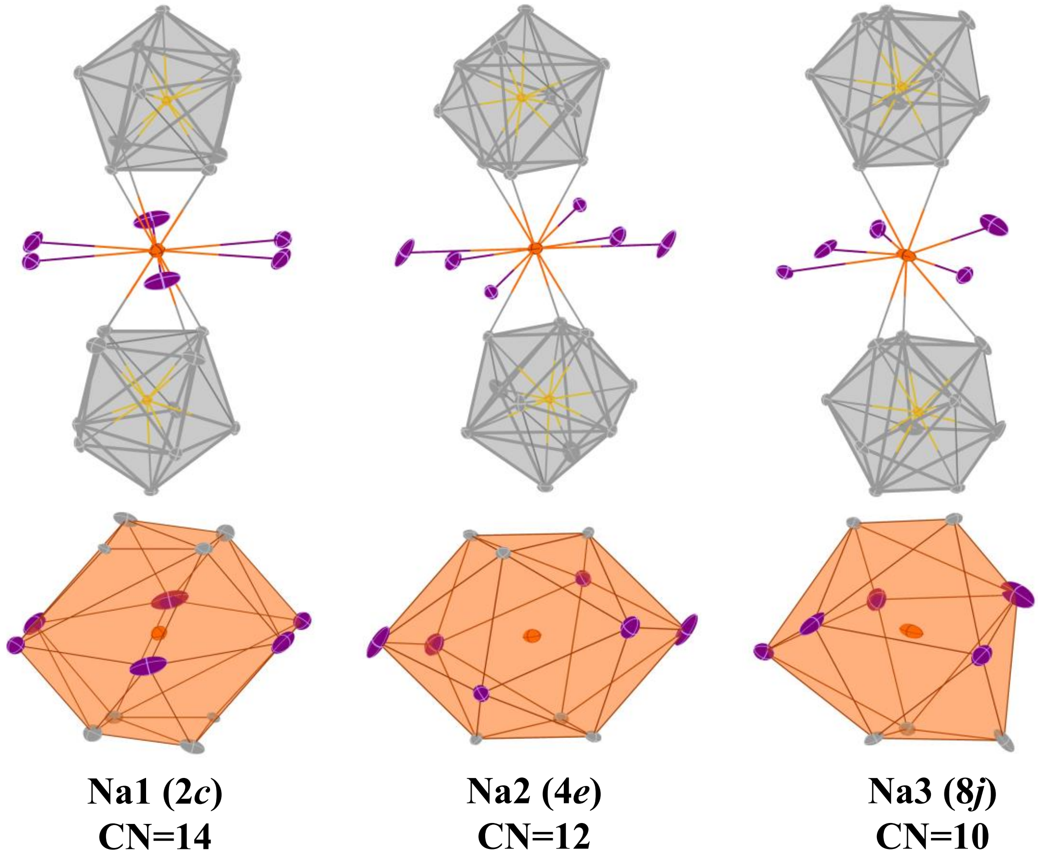

Figure S 18. *Coordination spheres of the three crystallographic independent sodium atoms in Na$_3$A$_6$In$_{10}$Au (A=Rb, Cs). The coordination number of Na3(8j) is the smallest, whereas Na1 (2c) and Na2 (4e) show coordination numbers of 14 and 12, respectively, which are often observed for sodium in intermetallic compounds.*

The surrounding of the three sodium atoms is very similar. All of them coordinate two $[Au@In_{10}]^{9-}$ clusters. Na3 (8*j*) has 10 neighbouring atoms (5xRb and 5xIn) in its coordination sphere and therefore the smallest coordination number (CN). One endohedral cluster is coordinated over an edge and the other one shows a μ^3 coordination. Na1 (2*c*) and Na2 (4*e*) both exhibit six rubidium atoms in their coordination sphere, which are orientated in an almost planar six membered ring around them. Underneath and above this planar arrangement the two $[Au@In_{10}]^{9-}$ clusters are located. Na2 shows a μ^3 coordination of the two clusters, whereas for Na1 both clusters are μ^4 coordinated. Thus, Na1 has the biggest CN of the three crystallographic independent sodium atoms. The Na-In (from 3.1 Å up to 3.4 Å) as well as the Na-Rb (from 3.8 Å up to 4.4 Å) and Na-Cs (from 3.52 Å up to 4.18 Å) distances respectively are all comparable with literature known values.[1–3] In Na_7RbIn_4 the Na-In distances range between 3.16 Å until 3.51 Å and in $Na_{26}Rb_3In_{48}$ corresponding values of 3.23 Å up to 3.49 Å are reported.[3] In Na_7RbIn_4 the Rb-Na distances reach from 3.67 Å up to 4.16 Å and in $Na_{26}Rb_3In_{48}$ corresponding distances between 3.94 Å and 4.13 Å are observed.[3]

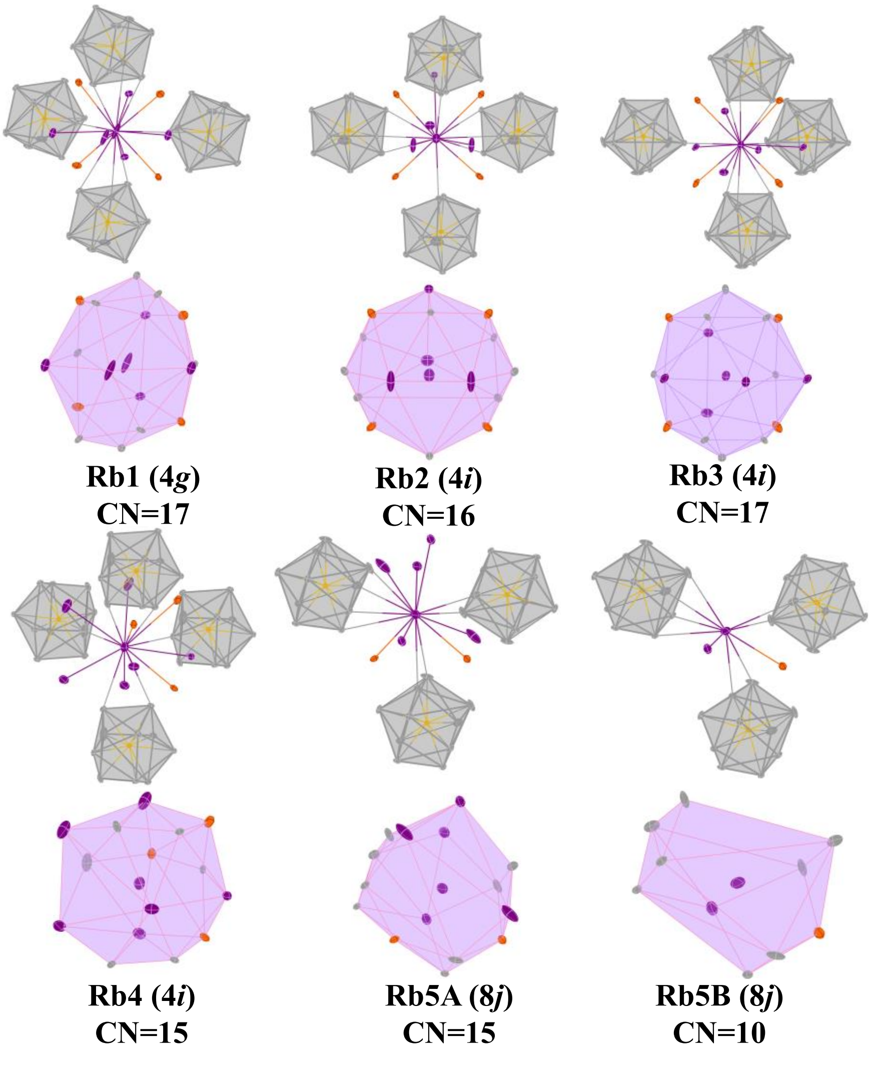

Figure S 19. *Coordination spheres of the six crystallographic independent rubidium atoms in* $Na_3Rb_6In_{10}Au$. *Rb5B shows relatively small coordination numbers for rubidium, whereas the other six rubidium atoms have coordination numbers between 15 and 17, which are often observed for rubidium in intermetallic compounds. For the cesium atoms in* $Na_3Cs_6In_{10}Au$ *the same coordination spheres and numbers are observed.*

Rb1 to Rb4, which are all fully occupied, coordinate each for $[Au@In_{10}]^{9-}$ clusters in different coordination modes. The two under occupied rubidium atoms, Rb5A and Rb5B, however only coordinate three endohedral $[Au@In_{10}]^{9-}$ clusters. Thus, they show smaller CN. This is especially true for Rb5B.

Rb1 (4*g*) exhibits a coordination number of 17, as it coordinates two $[Au@In_{10}]^{9-}$ clusters in an end-on fashion and the other two $[Au@In_{10}]^{9-}$ clusters are μ^3 coordinated. Further, Rb1 has four sodium as well as five rubidium atoms in its first coordination sphere. The coordination sphere of Rb2 (4*i*) and Rb3 (4*i*) are very similar to the one of Rb1, as all of them coordinate four $[Au@In_{10}]^{9-}$ clusters in total, in the same coordination mode, as well as four sodium atoms. The only difference is the number of rubidium atoms in their coordination sphere. Rb3 also coordinates five rubidium atoms and therefore exhibits a CN of 17, like Rb1 does. Rb2 only has four rubidium atoms in its coordination sphere and thus shows a reduced CN of 16. Rb4 (4*i*) coordinates four $[Au@In_{10}]^{9-}$ clusters, but two of them in an end-on fashion and the other two are μ^2 coordinated. Further, Rb4 only has three sodium atoms in its first coordination sphere as well as six rubidium atoms. Therefore, the CN of Rb4 is 15 and the smallest of the four fully occupied rubidium atoms. Rb5A and Rb5B (both 8*j*) coordinate three $[Au@In_{10}]^{9-}$ clusters, one of them with a μ^4 coordination mode and the other two with a μ^2 coordination mode. Rb5A further coordinates two sodium and five rubidium atoms, which gives a CN of 15. For Rb5B however only one sodium and one rubidium atom can be found in the first coordination sphere, which results in a comparable small CN of 10. For these coordination environments all these coordination environments the In-Rb distances are around 3.80 Å to 4.22 Å, the Rb-Na distances range from 3.45 Å to 4.17 Å and the Rb-Rb distances from 3.98 Å to 4.77 Å. Thus, they are again comparable to literature data as in Na_7RbIn_4 the In-Rb distances reach from 3.90 Å to 4.39 Å and the Rb-Na distances from 3.67 Å to 4.16 Å and in $Na_{26}Rb_3In_{48}$ the In-Rb distances range from 3.94 Å to 4.13 Å and the Rb-Na distances from 3.94 Å to 4.13 Å.

7.3 Splitting of Rb5 in $Na_3Rb_6In_{10}Au$

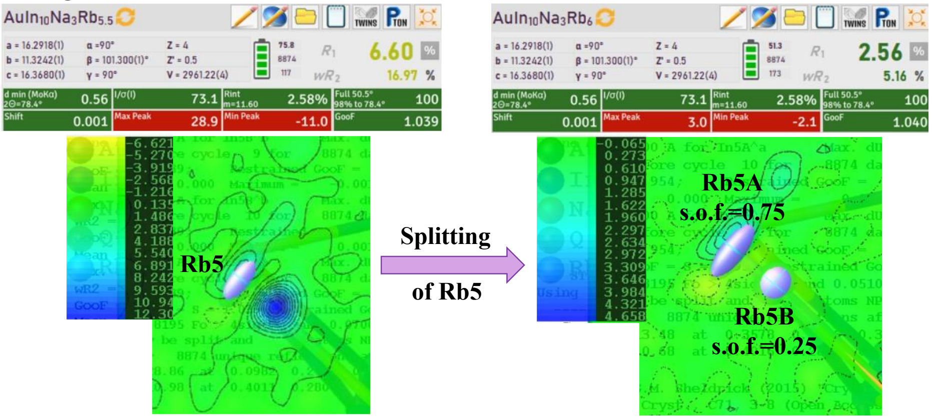

Figure S 20. *Residual electron density map around Rb5. On the left side Rb5 is not yet split. There, a big residual electron density peak can be located next to Rb5. When Rb5 is then split in Rb5A and Rb5B the high residual electron density disappears, the R values improve and the right sum formular is reached.*

As can be seen for example in Figure S20 alkali metal position of Rb5 (8*j*) is split into Rb5A and Rb5B with different coordination numbers. This is also supported by the residual electron density map. In *Olex* the Max. Peak is located next to Rb5 and further the stoichiometry is not right. When Rb5 is then split into two atoms Rb5A and Rb5B this Max. Peak disappears and the correct stoichiometry of $Na_3Rb_6In_{10}Au$ is obtained. For the split rubidium atoms an anisotropic refinement was appropriate as can be seen from the quality factors in Table 7.

Table S 7. *Comparison of the R-values, residual electron density and Goodness of fit (GOOF) for the isotropic refinement of the split rubidium atoms compared to anisotropic refinement.*

	**Isotropic refinement of split Rb**	**Anisotropic refinement of split Rb**
$\boldsymbol{R_1}$	5.51%	2.63%
$\boldsymbol{wR_2}$	13.64%	5.37%
**Min. Peak**	-11.9	-3.6
**Max. Peak**	17.3	3.2
**GOOF**	1.020	1.038

7.4 X-Ray Structure solution

Careful revision of the electron density at the indium positions did not show discrete positions, which would suggest split position model (see Figure S21). In analogy, rigid body refinement did not converge, while the (not senseful though) split model applied (see Figure S22) yielded no distinct positions and worse residual electron density (see Table S8). Therefore, the anharmonic refinement (see Figure S23) was chosen as final model as it describes the observed electron density of the compound best.

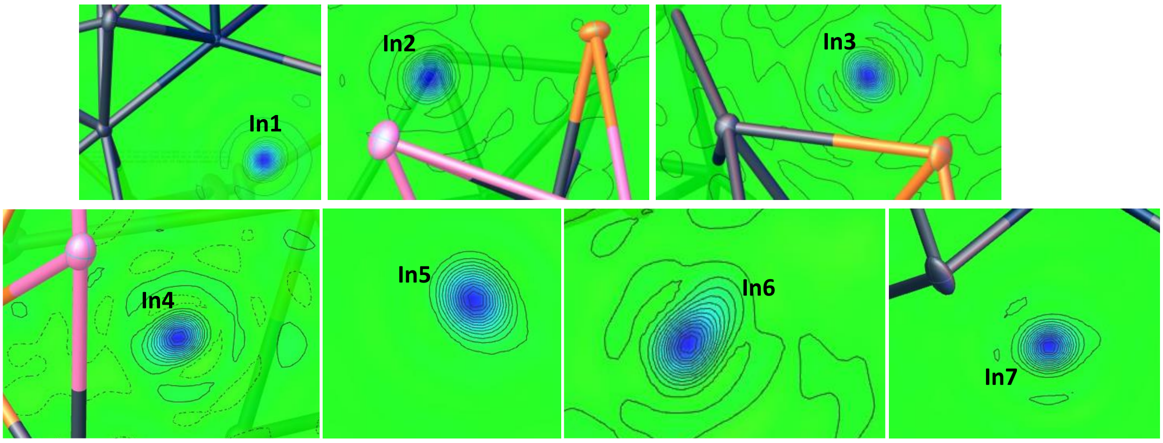

Figure S 21. *Here, the electron density maps of all indium positions are shown. While In1, In2 and In3 show a very symmetric electron density, In4, In5, In6 and In7 is more unsymmetric and distorted in one direction. This leads to the conclusion, that these positions may need "special treatment" such as splitting or better anharmonic refinement as discussed below.*

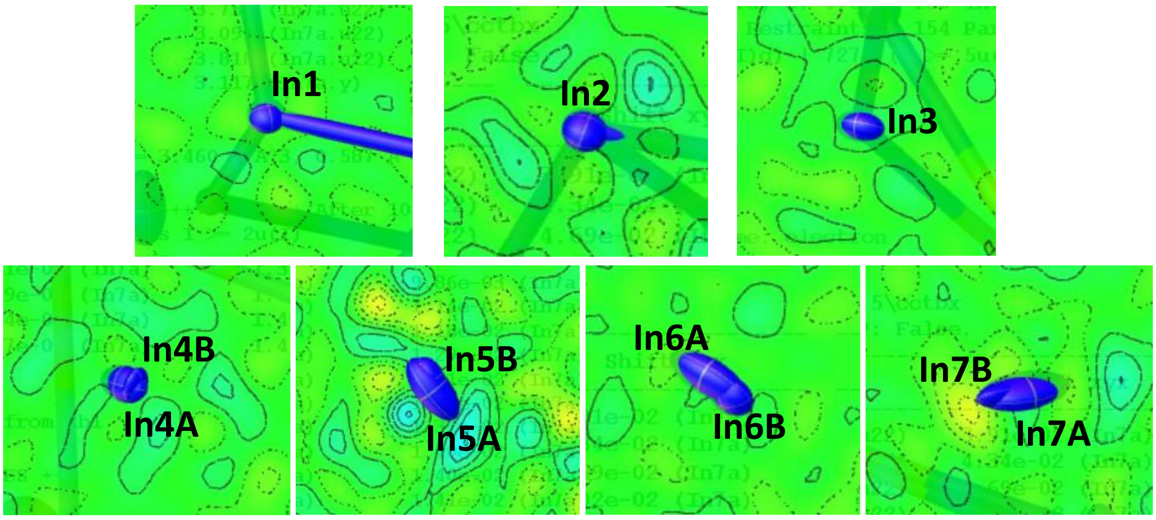

Figure S 22. *Here, the electron density maps of all indium positions are shown. The four indium positions In4A/B, In5A/, In6A/B and In7A/B were split but they move so close together that a split model is not very reasonable. Nothingness, the residual electron density is reduced, when the indium atoms are split but the shift does not fall. Therefore, we tried in the next step, to fix the atomic positions of the split indium atoms. That lead to slightly better values and the shift dropped.*

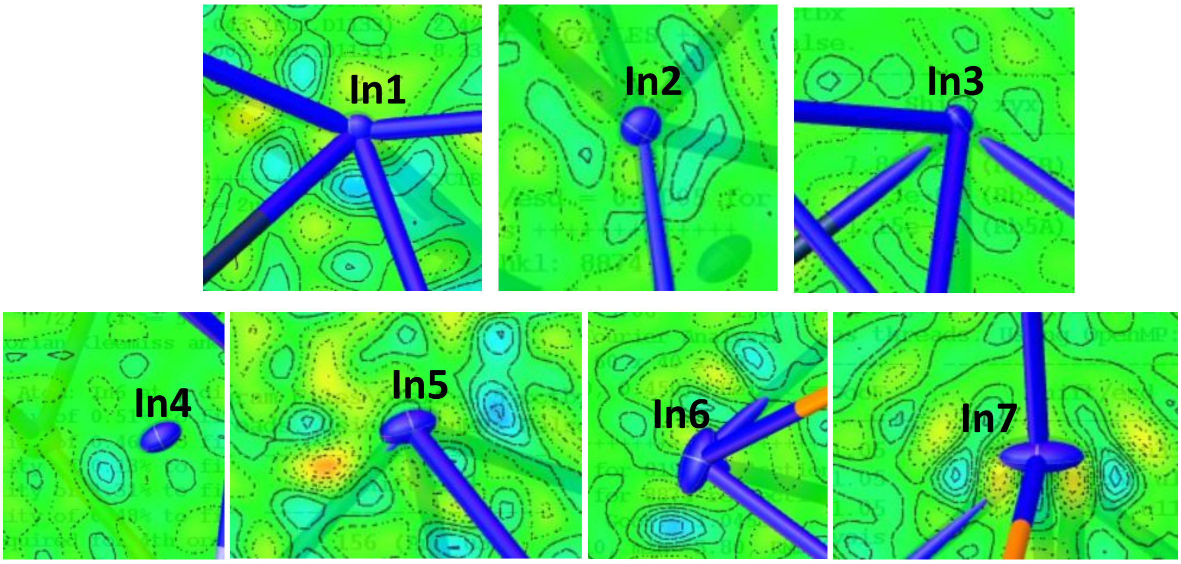

Figure S 23. *Here, the electron density maps of all indium positions are shown. After anharmonic refinement of In4, In5, In6, In7 the residual electron density is reduced. Therefore, we decided on this model.*

Table S 8. *Comparison of the R-values, residual electron density and Goodness of fit (GOOF) for the different models, which could be applied to interpret the X-Ray data.*

	**Without split position**	**Split position**	**Anharmonic refinement**
$\boldsymbol{R_1}$	4.73%	2.84%	3.39%
$\boldsymbol{wR_2}$	9.80%	5.86%	6.78%
**Min. Peak**	-11.0	-3.7	-3.4
**Max. Peak**	14.3	3.5	2.2
**GOOF**	1.013	1.042	1.042

In Table S 9 the quality factors for the isotropic refinement of the disordered atoms are compared. First, only for the disordered cesium atoms an isotropic refinement was carried out. Then the disordered

indium atoms were refined isotropically. Further, for both disordered atom types, cesium and indium, the isotropic refinement was carried out. This gave the worst quality factors compared to the anharmonic refinement.

Table S 9. *Comparison of the R-values, residual electron density and Goodness of fit (GOOF) for the isotropic refinement of the split cesium atoms, the split indium atoms and both, split cesium and indium atoms.*

	**Isotropic refinement of Cs**	**Isotropic refinement of In**	**Isotropic refinement of Cs and In**	**Anharmonic refinement**
R_1	3.59%	3.80%	4.28%	3.39%
wR_2	7.66%	8.13%	8.46%	6.78%
**Min. Peak**	-6.8	-6.4	-6.4	-3.4
**Max. Peak**	4.4	7.9	8.4	2.2
**GOOF**	1.069	1.065	1.045	1.042

8. Details of the Crystal Structure of $Na_{3.25}Cs_{5.75}In_{10}Au$

8.1 Unit cell and packing sequence of the anionic entities

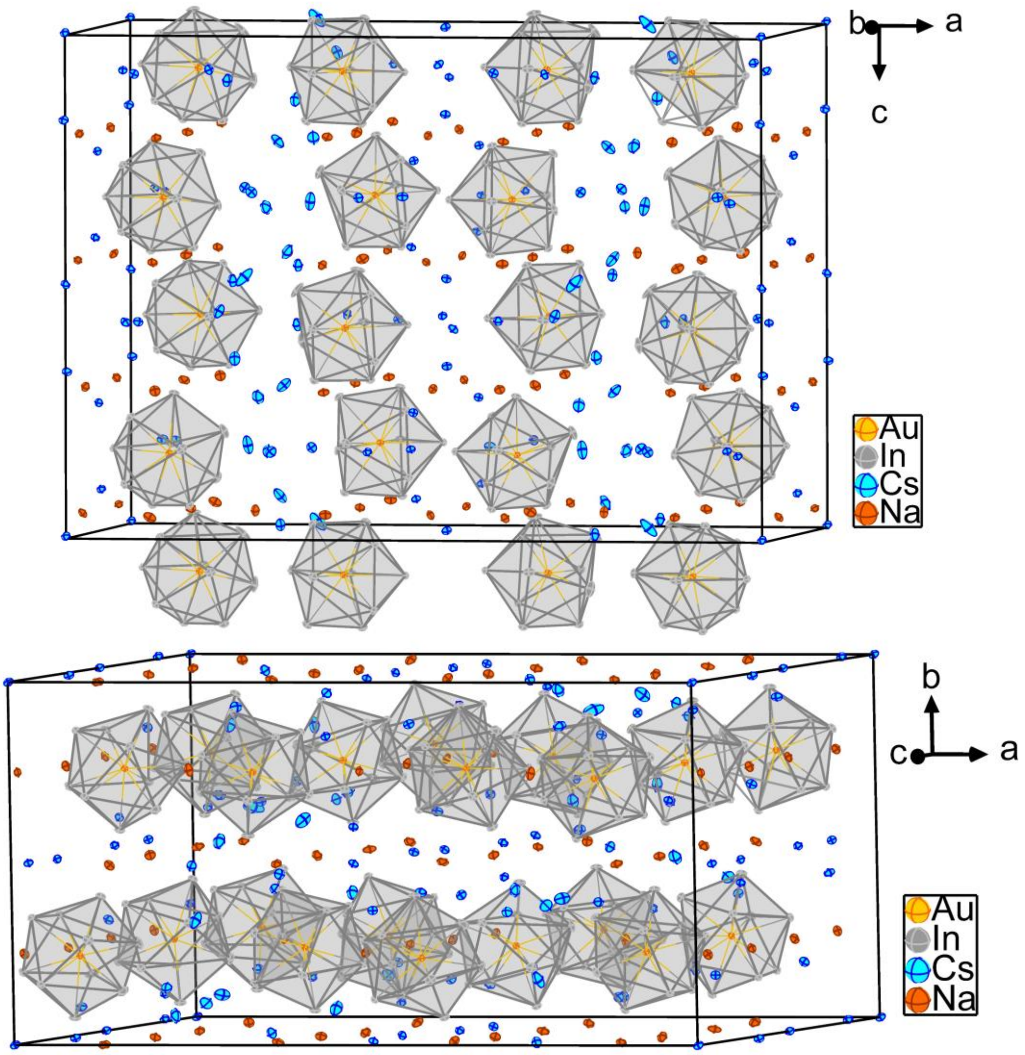

Figure S 24. *Unit cell of the compound $Na_{3.25}Cs_{5.75}In_{10}Au$ in the crystallographic b- (above) and c- (below) direction in the non-centrosymmetric space group $Pna2_1$.*

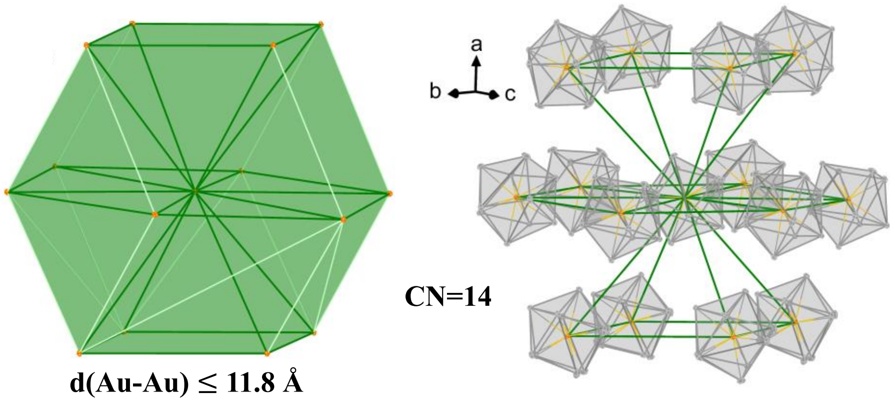

Figure S 25. *Around one $[Au@In_{10}]^{9-}$ clusters in $Na_{3.25}Cs_{5.75}In_{10}Au$ 14 further $[Au@In_{10}]^{9-}$ clusters. They do not form a densest packing but a Frank-Kasper polyhedron. The centers of gravity of the clusters, which correspond to the Au atoms, show distances underneath 11.8 Å.*

8.2 The four crystallographic independent $[Au@In_{10}]^{9-}$ clusters in $Na_{3.25}Cs_{5.75}In_{10}Au$

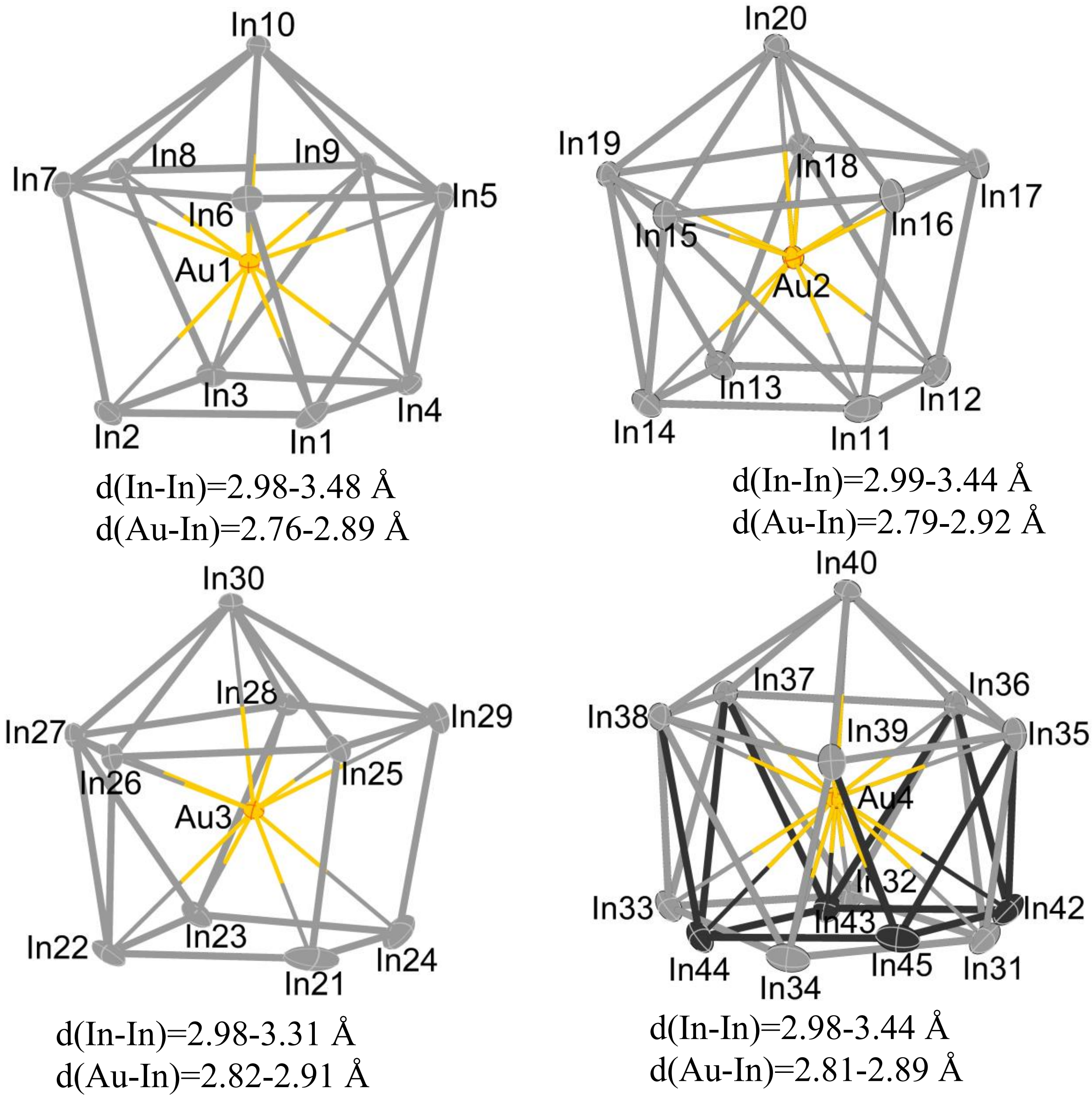

Figure S 26. *The four crystallographic independent* $[Au@In_{10}]^{9-}$ *clusters in* $Na_{3.25}Cs_{5.75}In_{10}Au$. *The In-In distances as well as the Au-In distances are in the same range for all the clusters and comparable with the ones in the lighter* $Na_3Rb_6In_{10}Au$. *This shows that the alkali metals used do not have a great influence on the size of the cluster. For two* $[Au@In_{10}]^{9-}$ *clusters (around Au2 and Au4) the disorder could be partly resolved (s.o.f. 0.9390(7)/0.0610(7)).*

The $[Au@In_{10}]^{9-}$ clusters show a disorder, which could only be resolved for two clusters (around Au2 and Au4) (s.o.f. 0.9390(7)/0.0610(7)). The site occupancy factors correspond to the ones of the cesium disorder, which means a concerted whole structure disorder. Unfortunately, this could only be resolved for part of the structure still yielding errors attributed to this feature in the CIF file. Mixed occupancies of cesium and sodium could be excluded due significant too high observed Fourier maxima for the positions, cesium resides on. Therefore, the whole structure is unquestionable.

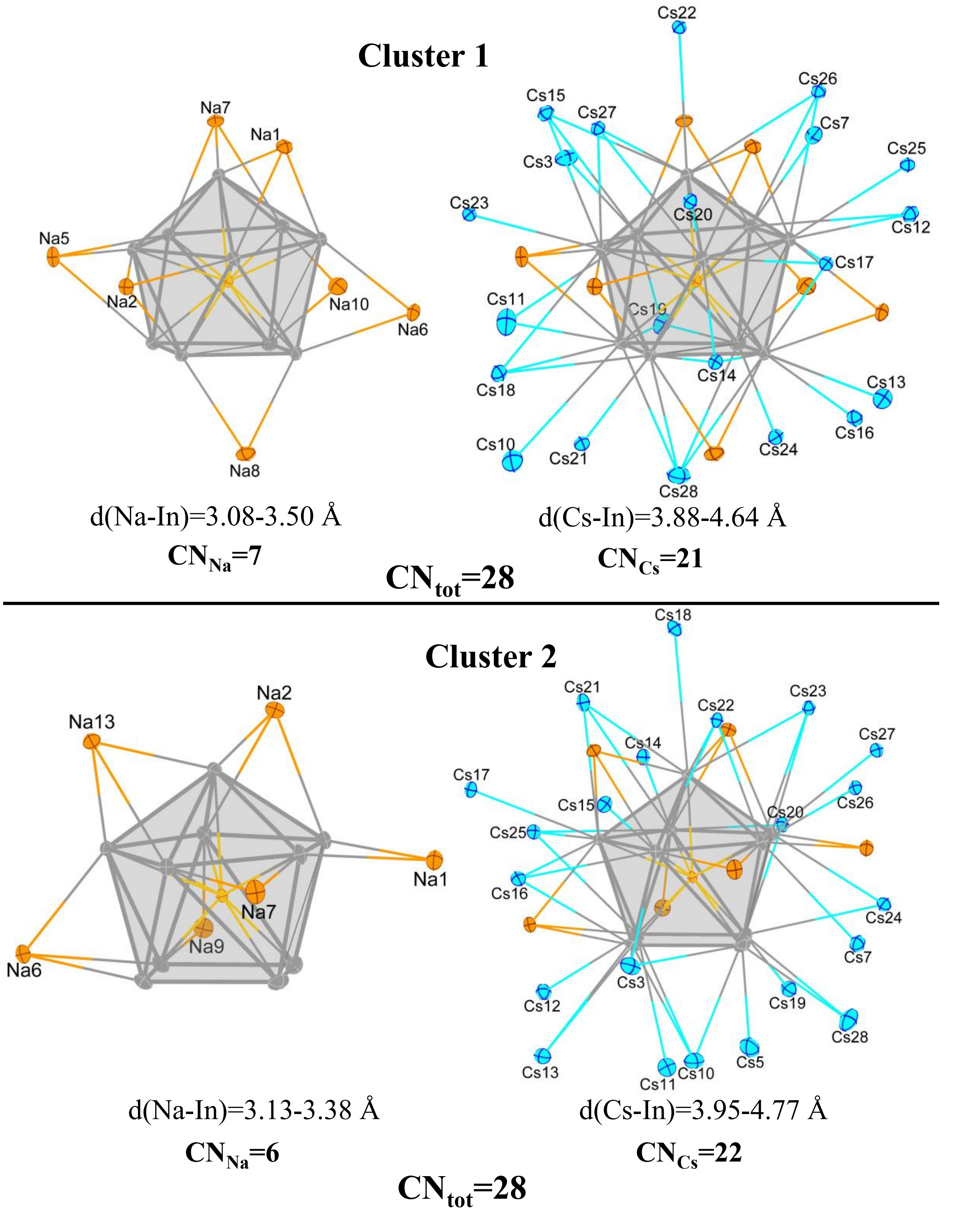

Figure S 27. *Coordination environment of Cluster 1 and Cluster 2 in $Na_{3.25}Cs_{5.75}In_{10}Au$. Both exhibit a coordination number of 28 alkali metals. Whereas Cluster 1 has seven sodium atoms but only 21 cesium atoms in its near surrounding, Cluster 2 shows six sodium atoms and 22 cesium atoms. Thereby, the In-Na distances are shorter than the In-Cs distances.*

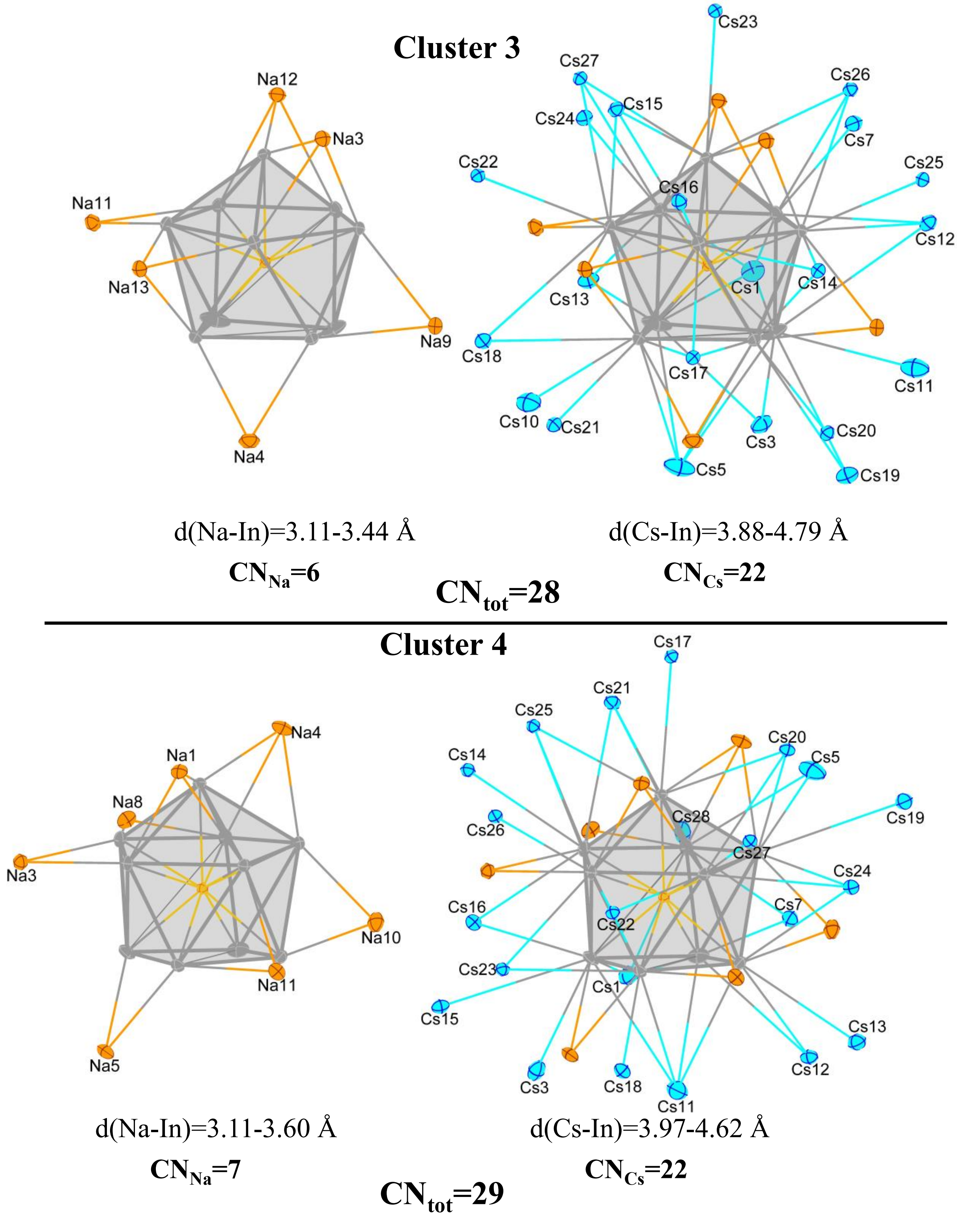

Figure S 28. *Coordination environment of Cluster 3 and Cluster 4 in* $Na_{3.25}Cs_{5.75}In_{10}Au$. *Cluster 3 exhibits also a coordination number of 28 alkali metals, with six sodium and 22 cesium atoms as it is observed for Cluster 1 and 2. Cluster 4 however is the only one with a coordination number of 29 as it is observed in* $Na_3Rb_6In_{10}Au$.

In $Na_{3.25}Cs_{5.75}In_{10}Au$ there are four crystallographic independent $[Au@In_{10}]^{9-}$ clusters. The In-In distances reach from 2.98 Å to 3.48 Å and are very similar in all for clusters. They are comparable to the In-In distances in the lighter homologue $Na_3Rb_6In_{10}Au$ as well as to reported data in literature. [4–7] The same is true for the In-Au distances, which range from 2.76 Å to 2.92 Å and are again comparable

to the same distances in $Na_3Rb_6In_{10}Au$ as well as to reported literature data. [8–12] This shows that the size of the alkali metal does not influence on the cluster size but on the resulting crystal structure.

The first three crystallographically independent $[Au@In_{10}]^{9-}$ clusters in the compound $Na_{3.25}Cs_{5.75}In_{10}Au$ have coordination numbers (CN) of 28, whereas the fourth cluster has a CN of 29 (see Figure S22). Here, cluster 2 und cluster 3 have six sodium atoms and 22 cesium atoms in their surroundings, cluster 1 coordinates seven sodium but only 21 cesium atoms. Clusters 4 however coordinates seven sodium but only 22 cesium atoms, what leads to a CN of 29 instead of 28. The Na-In (3.08 Å – 3.60 Å) as well as the Cs-In distances (3.88 Å – 4.79 Å) are comparable with literature data.[1,13–15]

9. SEM/EDS spectra

SEM/EDS spectra of the quaternary compounds $Na_3Rb_6In_{10}Au$ and $Na_{3.25}Cs_{5.75}In_{10}Au$ were measured to confirm the composition of the single crystal. Taking the errors into account the composition of the SEM/EDS spectra fits with the composition obtained from single crystal. Errors can be ascribed to a rough or not perfectly parallel orientation of the crystal surface. Due to the high sensitivity of the crystals against air and moisture decomposition might occur with only small traces of oxygen or moisture in the atmosphere. Slight amounts of carbon and oxygen can be ascribed to the sample carrier and minimal contamination due to possible leaks during the transport of the samples.

Table S 10. *Analysis of the EDS spectra of* $Na_3Rb_6In_{10}Au$.

Element	Atom Num.	Netto	Mass [%]	Mass Norm. [%]	Atom [%]	Abs. error [%] (1σ)	Rel. error [%] (1σ)
Na	11	7317	3.28	3.79	15.92	0.25	7.71
Rb	37	13027	20.53	23.72	26.82	0.62	3.00
In	49	239544	53.50	61.82	52.02	1.63	3.06
Au	79	13755	9.24	10.67	5.24	0.28	2.99

$Na_3Rb_6In_{10}Au$: atomic percentage calculated/measured: 15/15.92 (Na), 30/26.82(Rb), 50/52.02 (In), 5/5.24 (Au).

Table S 11. *Analysis of the EDS spectra of* $Na_{3.25}Cs_{5.75}In_{10}Au$.

Element	Atom Num.	Netto	Mass [%]	Mass Norm. [%]	Atom [%]	Abs. error [%] (1σ)	Rel. error [%] (1σ)
Na	11	3975	2.5	3.55	17.04	0.24	8.04
In	49	136906	40.27	48.54	46.61	1.24	3.08
Cs	55	69150	29.35	35.38	29.35	0.84	2.85
Au	79	10423	10.39	12.52	7.01	0.31	3.02

$Na_{3.25}Cs_{5.75}In_{10}Au$: atomic percentage calculated/measured: 17/17.04 (Na), 31/29.35 (Cs), 50/46.61 (In), 5/7.01 (Au).

10. ^{23}Na-Solid-State-NMR spectroscopy

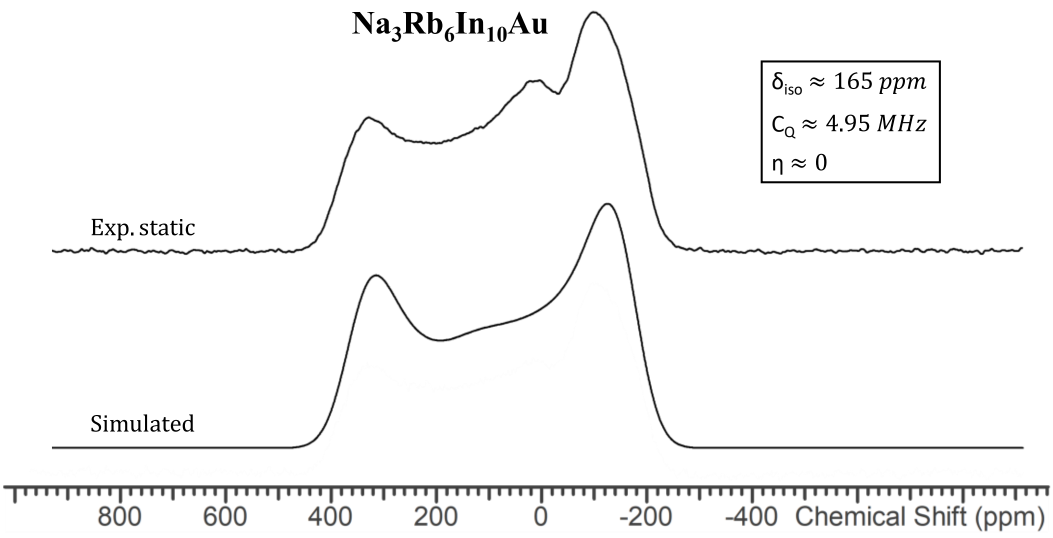

Figure S 29. *Measured (above) and simulated (below)* ^{23}*Na-NMR spectrum of the compound* $Na_3Rb_6In_{10}Au$*. Experimental details can be found in the Experimental Section and a detailed discussion is given in the main paper.*

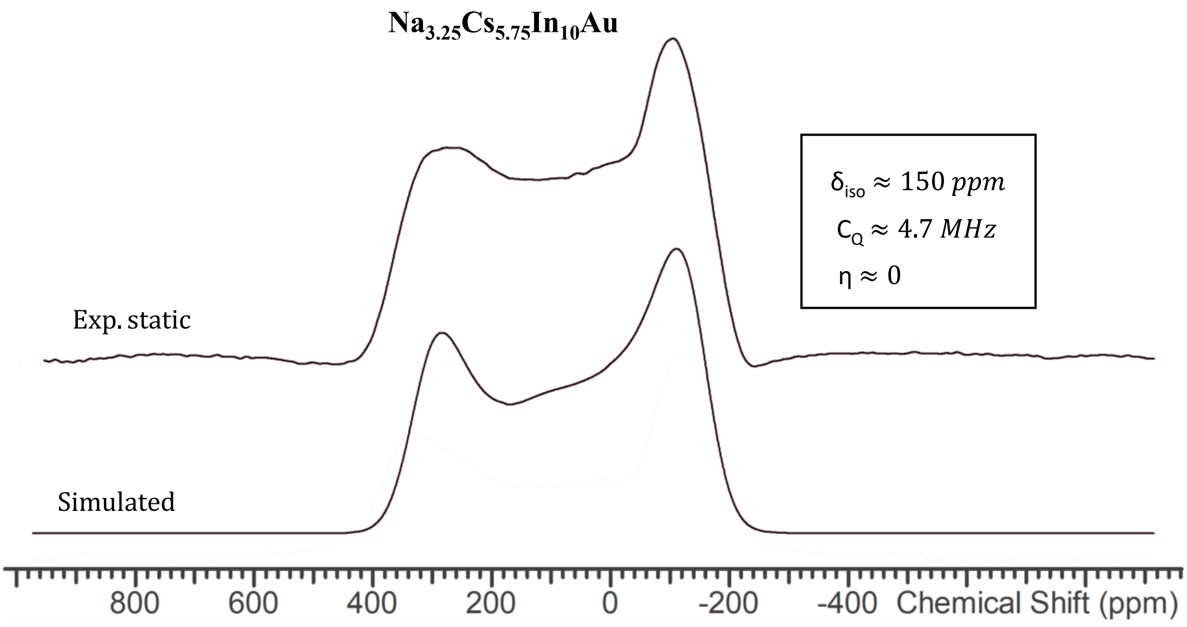

Figure S 30. *In the upper part the measured (above) and simulated (below)* ^{23}*Na-NMR spectrum of the compound* $Na_{3.25}Cs_{5.75}In_{10}Au$ *is shown. In the lower part the* ^{23}*Na-MAS spectrum can be seen as for* $Na_{3.25}Cs_{5.75}In_{10}Au$ *a slow spinning rate was possible. Experimental details can be found in the Experimental Section and a detailed discussion is given in the main paper.*

11. Theoretical calculations

11.1 In-Au Interactions in the Density of states

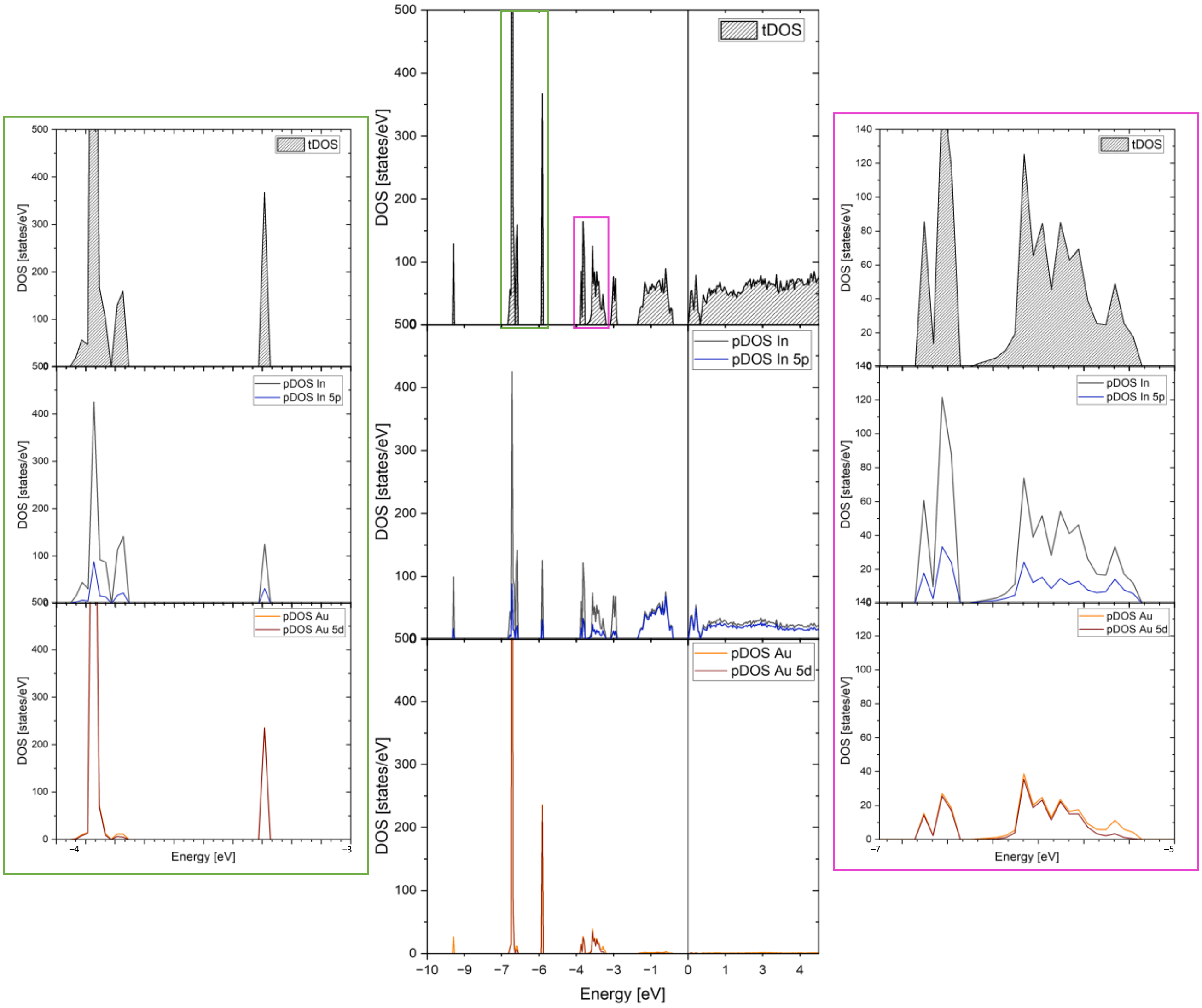

Figure S 31. *The interactions between gold and indium are represented by a high DOS from –4 eV to–3 eV (left, pink) as well as from –5.5 eV and –7 eV (right, green). These interactions take place between the 5d orbitals of the gold and the 5p orbitals of the indium.*

11.2 Scalar relativistic band structure plot of $Na_3Rb_6In_{10}Au$

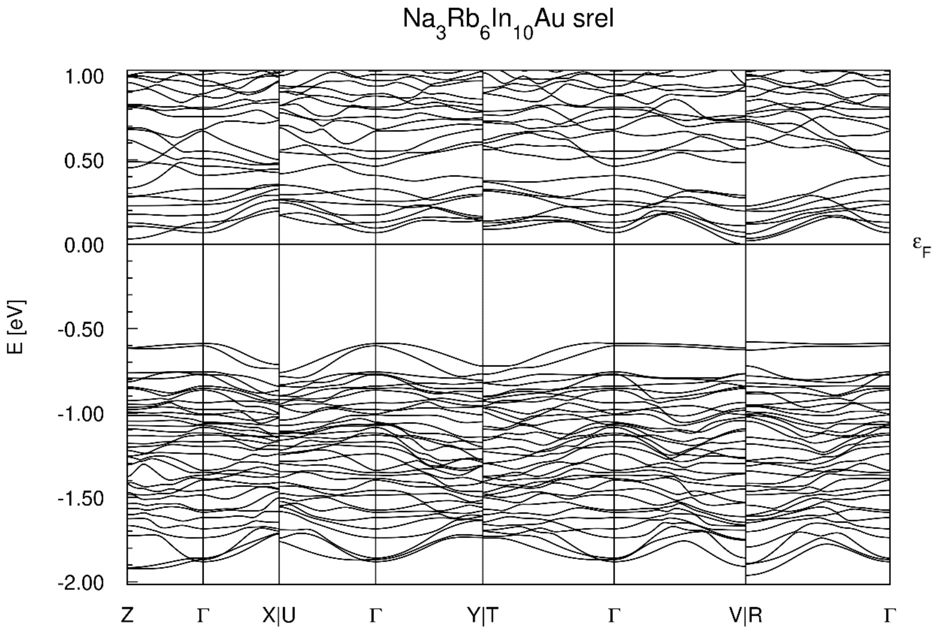

Figure S 32. *Scalar relativistic band structure plot of the compound $Na_3Rb_6In_{10}Au$. For the calculation an ordered model with fully occupied alkali metal positions was used (see CIF below). The indirect band gap* (Γ→V|R) *is around 0.51eV and no bands are cutting through the Fermi level.*

11.3 Full relativistic band structure plot of $Na_3Rb_6In_{10}Au$

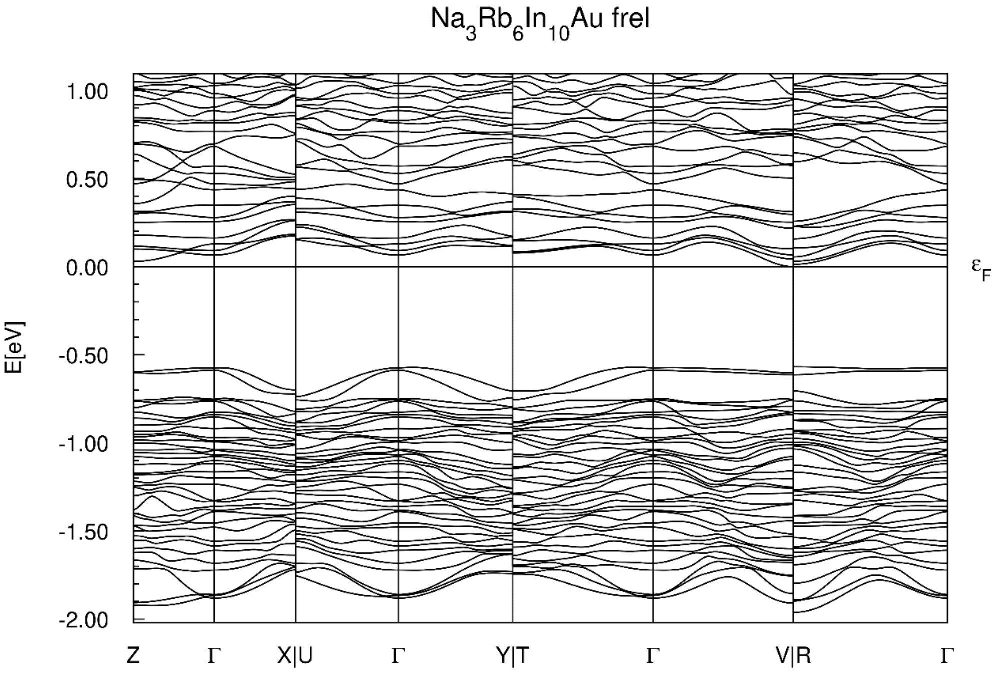

Figure S 33. *Full relativistic band structure plot of the compound $Na_3Rb_6In_{10}Au$. For the calculation an ordered model with fully occupied alkali metal positions was used (see CIF below). The indirect band gap* (Γ→V|R) *is around 0.50 eV and no bands are cutting through the Fermi level. Compared to the Scalar relativistic band structure plot the band gap is a little bit smaller.*

11.4 CIF file of the fully ordered mode for $Na_3Rb_6In_{10}Au$

```
_audit_creation_method 'Created with Endeavour Version 1.8b'
_audit_creation_date      2025-01-30
_audit_update_record      2025-01-30
_chemical_formula_sum     'Au In10 Rb5.454 Na2.591'
_chemical_formula_weight  1870.863
_refine_ls_R_factor_all   0.055
_cell_length_a     16.3056
_cell_length_b     11.3364
_cell_length_c     16.3778
_cell_angle_alpha  90.000
_cell_angle_beta   101.270
_cell_angle_gamma  90.000
_cell_volume 2969.0(0)
_cell_formula_units_Z     4
_symmetry_int_tables_number      2
_symmetry_space_group_name_H-M   'P -1'
_symmetry_space_group_name_Hall  '-P_1'

loop_
_symmetry_equiv_pos_site_id
_symmetry_equiv_pos_as_xyz
1 x,y,z
2 -x,-y,-z

loop_
_atom_type_symbol
_atom_type_oxidation_number
_atom_type_radius_bond
Au    ?     1.200
In    ?     1.200
Rb    ?     1.200
Na    ?     1.200


loop_
_atom_site_label
_atom_site_type_symbol
_atom_site_fract_x
_atom_site_fract_y
_atom_site_fract_z
_atom_site_occupancy
_atom_site_symmetry_multiplicity
_atom_site_Wyckoff_symbol
_atom_site_attached_hydrogens
_atom_site_calc_flag
_atom_site_thermal_displace_type
_atom_site_u_iso_or_equiv
Au1 Au  0.6891  0.5000  0.7544  1.000 2 i ? d ? ?
Au1 Au  0.1891  0.0000  0.7544  1.000 2 i ? d ? ?
In1 In  0.5124  0.5000  0.7034  1.000 2 i ? d ? ?
In1 In  0.0124  0.0000  0.7034  1.000 2 i ? d ? ?
In1 In  0.7703  0.7201  0.7982  1.000 2 i ? d ? ?
In1 In  0.2703  0.2201  0.7982  1.000 2 i ? d ? ?
In1 In  0.2297  0.7201  0.2018  1.000 2 i ? d ? ?
In1 In  0.7297  0.2201  0.2018  1.000 2 i ? d ? ?
In1 In  0.7864  0.5000  0.9228  1.000 2 i ? d ? ?
In1 In  0.2864  0.0000  0.9228  1.000 2 i ? d ? ?
In1 In  0.6096  0.6355  0.8647  1.000 2 i ? d ? ?
In1 In  0.1096  0.1355  0.8647  1.000 2 i ? d ? ?
In1 In  0.3904  0.6355  0.1353  1.000 2 i ? d ? ?
In1 In  0.8904  0.1355  0.1353  1.000 2 i ? d ? ?
In1 In  0.8597  0.5000  0.7478  1.000 2 i ? d ? ?
In1 In  0.3597  0.0000  0.7478  1.000 2 i ? d ? ?
In1 In  0.6200  0.2989  0.6604  1.000 2 i ? d ? ?
In1 In  0.1200  0.7989  0.6604  1.000 2 i ? d ? ?
In1 In  0.3800  0.2989  0.3396  1.000 2 i ? d ? ?
```

In1	In	0.8800	0.7989	0.3396	1.000	2	i	?	d	?	?
In1	In	0.7139	0.5000	0.5908	1.000	2	i	?	d	?	?
In1	In	0.2139	0.0000	0.5908	1.000	2	i	?	d	?	?
Rb1	Rb	0.6197	0.0000	0.8180	1.000	2	i	?	d	?	?
Rb1	Rb	0.1196	0.5000	0.8180	1.000	2	i	?	d	?	?
Rb1	Rb	0.5000	0.8286	0.0000	1.000	2	i	?	d	?	?
Rb1	Rb	0.0000	0.3286	0.0000	1.000	2	i	?	d	?	?
Rb1	Rb	0.3902	0.5000	0.9074	1.000	2	i	?	d	?	?
Rb1	Rb	0.8902	0.0000	0.9074	1.000	2	i	?	d	?	?
Rb1	Rb	0.7335	0.5000	0.3552	1.000	2	i	?	d	?	?
Rb1	Rb	0.2335	0.0000	0.3552	1.000	2	i	?	d	?	?
Rb1	Rb	0.3826	0.7883	0.5575	1.000	2	i	?	d	?	?
Rb1	Rb	0.8827	0.2883	0.5575	1.000	2	i	?	d	?	?
Rb1	Rb	0.6173	0.7883	0.4425	1.000	2	i	?	d	?	?
Rb1	Rb	0.1173	0.2883	0.4425	1.000	2	i	?	d	?	?
Na1	Na	0.7500	0.7500	0.0000	1.000	2	i	?	d	?	?
Na1	Na	0.2500	0.7500	0.0000	1.000	2	i	?	d	?	?
Na1	Na	0.5000	0.5000	0.5000	1.000	1	h	?	d	?	?
Na1	Na	0.0000	0.0000	0.5000	1.000	1	b	?	d	?	?
Na1	Na	0.4547	0.7524	0.7697	1.000	2	i	?	d	?	?
Na1	Na	0.9547	0.2524	0.7697	1.000	2	i	?	d	?	?
Na1	Na	0.0453	0.2524	0.2303	1.000	2	i	?	d	?	?

References

(1) Janesch, M.; Gjorgjevikj, K.; Krause, S.; Gärtner, S. Crystal Structure of Na_7RbIn_4 and the solid solutions $Na_7K\mathit{Tr}_4$ and $Na_7Rb\mathit{Tr}_4$ (*Tr*=In, Tl). *Z. Kristallogr. Crys. Mat.* **2025**, in preparation.

(2) Sevov, S. C.; Corbett, J. D. Synthesis, characterization, and bonding of indium clusters. Rb_2In_3, a Zintl phase with layers of *closo*-indium octahedra. *Z. Anorg. Allg. Chem.* **1993**, *619* (1), 128–132. DOI: 10.1002/zaac.19936190121.

(3) Sevov, S. C.; Corbett J. D. Synthesis, Characterization, and Bonding of Indium Clusters: $A_3Na_{26}In_{48}$ (*A*=K, Rb, Cs) with a Novel Cubic Network of *arachno*- and *closo*-In12 Clusters. *Inorg. Chem.* **1993** (32), 1612–1615.

(4) Sevov, S. C.; Corbett, J. D. A remarkable hypoelectronic indium cluster in K_8In_{11}. *Inorg. Chem.* **1991**, *30* (26), 4875–4877. DOI: 10.1021/ic00026a004.

(5) Sevov, S. C.; Corbett, J. D. $K_{10}In_{10}Z$ (*Z* = Ni, Pd, Pt): Zintl phases containing isolated decaindium clusters centered by transition elements. *J. Am. Chem. Soc.* **1993**, *115* (20), 9089–9094. DOI: 10.1021/ja00073a026.

(6) Saltykov, V.; Nuss, J.; Wedig, U.; Prasad, D. L. V. K.; Jansen, M. First Isolated "Hypoelectronic" $[In_6]^{6-}$ Cluster in Insulating $Cs_{22}In_6(SiO_4)_4$. *Z. Anorg. Allg. Chem.* **2011**, *7-8* (637), 834–839. DOI: 10.1002/zaac.201100074.

(7) Janesch, M.; Schwinghammer, V. F.; Shenderovich, I. G.; Gärtner, S. Synthesis and characterization of ternary trielides $Na_7K\mathit{Tr}_4$ [*Tr* =In or Tl] including $[\mathit{Tr}_4]^{8-}$ Tetrahedra. *Z. Anorg. Allg. Chem.* **2023**, *649* (21). DOI: 10.1002/zaac.202300112.

(8) Gabbai, F. P.; Schier, A.; Riede, J.; Schmidbaur, H. Different Pathways of the Reaction of InCl with Ph_3PAuCl: Isolation of the First Mixed-Valent Mixed-Metal Gold/Indium Cluster. *Inorg. Chem.* **1995**, *34* (15), 3855–3856. DOI: 10.1021/ic00119a003.

(9) Gabbaï, F. P.; Chung, S.-C.; Schier, A.; Krüger, S.; Rösch, N.; Schmidbaur, H. A Novel Anionic Gold-Indium Cluster Compound: Synthesis and Molecular and Electronic Structure. *Inorg. Chem.* **1997**, *36* (25), 5699–5705. DOI: 10.1021/ic970725c.

(10) Zachwieja, U. $NaAuIn_2$, ein ternäres Aurid mit ethananalogen In_2Au_6-Baueinheiten und $[In_{2/2}]$-Ketten. *Z. Anorg. Allg. Chem.* **1995**, *621* (10), 1677–1680.

(11) Zachwieja, U. $Na_2Au_6In_5$, the first compound in the system sodium-gold-indium. *J. Alloys Comp.* **1996**, *235* (1), 7–11. DOI: 10.1016/0925-8388(95)02113-2.

(12) Zachwieja, U. $Na_8Au_{11}In_6$: ein Gold-Indium-Polyedergerüst mit pentagonal-bipyramidalen $AuAu_5In$-Baueinheiten. *Z. Anorg. Allg. Chem.* **1996**, *622* (9), 1581–1586.

(13) Yatsenko, S. P.; Tschuntonow, K. A.; Orlow, A. N.; Yarmolyuk, Y.; Hryn, Y. Kristallstruktur von Cs_2In_3. *J. Less Common Met.* **1985**, *108* (2), 339–343. DOI: 10.1016/0022-5088(85)90228-0.

(14) Sevov, S. C.; Corbett, J. D. Synthesis, characterization, and bonding of indium clusters: $A_3Na_{26}In_{48}$ (*A* = K, Rb, Cs) with a novel cubic network of *arachno*- and *closo*-In_{12} clusters. *Inorg. Chem.* **1993**, *32* (9), 1612–1615. DOI: 10.1021/ic00061a016.

(15) Janesch, M.; Karttunen, A. J.; Gärtner, S. Icosahedral Clusters $[In@\mathit{Tr}_{12}]^{10-}$: Synthesis, Characterization, and Electronic Structure Investigations of $Na_4A_6Tr_{13}$ (*A* = Rb, Cs; *Tr* = In, Tl). *Eur. J. Inorg. Chem.* **2025**, e202500347. DOI: 10.1002/ejic.202500347.
